# Evidence for Host-Genotype Associations of *Borrelia burgdorferi* Sensu Stricto

**DOI:** 10.1371/journal.pone.0149345

**Published:** 2016-02-22

**Authors:** Samir Mechai, Gabriele Margos, Edward J. Feil, Nicole Barairo, L. Robbin Lindsay, Pascal Michel, Nicholas H. Ogden

**Affiliations:** 1 Groupe de Recherche en Épidémiologie des Zoonoses et Santé Publique, Faculté de Médecine Vétérinaire, Université de Montréal, Saint-Hyacinthe, Québec, Canada; 2 National Reference Centre for Borrelia, Oberschleissheim, Germany; 3 Bavarian Health and Food Safety Authority, Oberschleissheim, Germany; 4 The Milner Centre for Evolution, Department of Biology and Biochemistry, University of Bath, Claverton Down, Bath, United Kingdom; 5 National Microbiology Laboratory, Public Health Agency of Canada, Winnipeg, Manitoba, Canada; 6 National Microbiology Laboratory, Public Health Agency of Canada, Saint-Hyacinthe, Québec, Canada; University of Kentucky College of Medicine, UNITED STATES

## Abstract

Different genotypes of the agent of Lyme disease in North America, *Borrelia burgdorferi* sensu stricto, show varying degrees of pathogenicity in humans. This variation in pathogenicity correlates with phylogeny and we have hypothesized that the different phylogenetic lineages in North America reflect adaptation to different host species. In this study, evidence for host species associations of *B*. *burgdorferi* genotypes was investigated using 41 *B*. *burgdorferi*-positive samples from five mammal species and 50 samples from host-seeking ticks collected during the course of field studies in four regions of Canada: Manitoba, northwestern Ontario, Quebec, and the Maritimes. The *B*. *burgdorferi* genotypes in the samples were characterized using three established molecular markers (multi-locus sequence typing [MLST], 16S-23S *rrs-rrlA* intergenic spacer, and outer surface protein C sequence [*ospC*] major groups). Correspondence analysis and generalized linear mixed effect models revealed significant associations between *B*. *burgdorferi* genotypes and host species (in particular chipmunks, and white-footed mice and deer mice), supporting the hypotheses that host adaptation contributes to the phylogenetic structure and possibly the observed variation in pathogenicity in humans.

## Introduction

In North America, *Borrelia burgdorferi* sensu stricto (hereafter termed *B*. *burgdorferi* for simplicity) is a member of the bacterial genospecies complex *B*. *burgdorferi* sensu lato (s.l.) that is associated with Lyme disease [1]. In Eurasia, five genospecies of the *B*. *burgdorferi* s.l. complex are associated with Lyme disease [1]: *B*. *burgdorferi*, *B*. *afzelii*, *B*. *garinii*, *B*. *bavariensis* and *B*. *spielmanii* and the two main tick vectors are *Ixodes ricinus* (in Europe) and *I*. *persulcatus* (in Asia) [2, 3]). In North America, *B*. *burgdorferi* is mostly transmitted by two tick species: *I*. *scapularis* in the regions encompassing northeastern USA and southeastern Canada, and the upper Midwest USA and south central Canada, and *I*. *pacificus* in the western coastal states of the USA and in British Columbia, Canada.

In Eurasia, the different *B*. *burgdorferi* s.l. genospecies are associated with different types of clinical disease [4]. Arthritis is associated with *B*. *burgdorferi* infection; neuroborreliosis with *B*. *garinii* and *B*. *bavariensis* infection, and chronic dermatological manifestations with *B*. *afzelii* [5–7]. Most of the clinical features seen in Europe are also seen in North America, and these include those of early Lyme disease (Erythema migrans: EM), early disseminated Lyme disease (neuroborreliosis including facial palsy, meningitis and peripheral radiculoneuropathy, and atrioventricular block) and late disseminated Lyme disease (including Lyme arthritis) [2, 8, 9]. In North America there is evidence that different genotypes of *B*. *burgdorferi* show different levels of pathogenicity in humans, specifically whether or not the bacterium disseminates systemically from the early phase infection in the skin where the infective tick bit the patient [10–14]. In Europe, *B*. *burgdorferi* s.l. genospecies are frequently specialized for transmission by different host species [15]: *B*. *afzelii* and *B*. *bavariensis* are rodent host specialists [15, 16], *B*. *garinii* is a bird specialist [8] and *B*. *lusitaniae* may be a lizard specialist [17]. In North America, *B*. *burgdorferi* is considered a host generalist [4, 18], although more stable suitable environments associated with expanding woodland habitats, increased abundance of tick vectors and reservoir hosts [19] are thought to be creating conditions favourable for adaptive radiation and multiple niche polymorphism [4]. Most parasites show some degree of host preference [20–22], which is a critical pre-adaptation for host specialization if conditions for transmission are suitable [23], as they may increasingly be for *B*. *burgdorferi* in North America. There is some evidence of host associations for *B*. *burgdorferi* in the form of unequal frequencies of *B*. *burgdorferi* genotypes in samples collected in the field from different sources [24–26], and differential infection and transmission efficiency among different host-genotype pairings [25, 27]. Such associations are of public health interest as they may be linked to the capacity of the different genotypes to show different pathogenicity in humans and varying capacity to stimulate antibodies detectable in current serological tests, while the existence of host associations may allow prediction of regions and habitats where different genotypes are more likely to occur [28].

The clade structure of the *B*. *burgdorferi* phylogenetic tree obtained using concatenated housekeeping genes of a multilocus sequence typing (MLST) method does not seem to be based on geographic isolation of genotypes [29, 30]. It has been hypothesized that the clades were associated with introductions and/or population expansions after bottlenecks possibly associated with glacial-interglacial periods [31], although ecological isolation driven by host species associations may also explain the origin and maintenance of discrete clusters [28]. Small and medium-sized vertebrates, particularly rodents, are key requirements for *B*. *burgdorferi* transmission cycles in northern North America as these species are frequently competent reservoirs of *B*. *burgdorferi* and important hosts for immature ticks. Adult ticks feed preferentially on larger mammals, mostly reservoir-incompetent deer [32]. The near absence of *B*. *burgdorferi* from *I*. *scapularis* ticks in southeastern USA is thought to be associated in part with the high proportion of immature ticks in this region that feed on reservoir-incompetent lizards and the low proportion that feed on reservoir-competent rodents [33].

Lyme disease is currently emerging in central and southeastern Canada associated with the northward expansion of the geographic range of *I*. *scapularis*, which is possibly associated with climate change [34, 35]. Range expansion of both *I*. *scapularis* and *B*. *burgdorferi* is likely being facilitated by dispersal of ticks and bacteria by both migratory birds and terrestrial hosts [36, 37]. It is also possible that refugial populations of *B*. *burgdorferi* are maintained by nidicolous ticks [38] but these have yet to be found in Canada.

A complex geographic pattern of genotypes has been found in emerging Lyme disease risk areas in south central and southeastern Canada [30], and in this study we explore possible association of *B*. *burgdorferi* genotypes with host species using samples collected in these regions.

## Material and Methods

### Samples used in the study

The samples used in this study comprise DNA of *B*. *burgdorferi* extracted from host-seeking *I*. *scapularis* ticks, engorged ticks from captured rodent hosts and from *B*. *burgdorferi*-positive tissues from rodent hosts. If there were multiple samples from the same rodent (which could be either an engorged immature tick or a tissue sample), only one sample per rodent host was randomly selected for inclusion in the study. All samples used in host association analyses in this study were collected during field studies in 41 woodland locations in south central and southeastern Canada from 2006 to 2013. In these studies rodents were captured using Sherman traps, examined for ticks under anaesthesia, and subsequently euthanized by cervical dislocation under anaesthesia. Feeding ticks and rodent tissues were collected and transferred to the laboratory for testing for *B*. *burgdorferi* as previously described [39, 40]. Host-seeking ticks were collected by drag sampling and also transferred to the laboratory for testing for *B*. *burgdorferi* [40]. Rodents were trapped on private properties that did not require specific permissions and did not involve the capture of endangered or protected species. All rodents were captured and dispatched using protocols approved by animal care committees of either the Canadian Science Centre for Human and Animal Health or Université de Montréal, and with relevant scientific collection permits for the locations where the work was conducted (Manitoba Conversation, Ontario Ministry of Natural Resources, Ministère des Ressources naturelles et de la Faune du Québec and the Nova Scotia Department of Natural Resources).

For the host association analyses in this study there was a total of 91 *B*. *burgdorferi*-positive samples from the sites in Canada including 41 samples from 5 rodent species namely: deer mouse (*Peromyscus maniculatus* Wagner, 1845, n = 2), eastern chipmunk (*Tamias striatus* Linnaeus, 1758, n = 16), red squirrel (*Tamiasciurus hudsonicus* Erxleben, 1777, n = 2), red backed vole (*Myodes gapperi* Vigors, 1830, n = 1) and white-footed mouse (*Peromyscus leucopus* Rafinesque, 1818, n = 20), as well as 50 questing ticks collected contemporaneously with the samples from rodents (Table 1). DNA was extracted from all samples (questing ticks, engorged ticks and host tissue samples) and screened for *B*. *burgdorferi* infection by polymerase chain reaction (PCR) as previously described [30, 36]. Sampling effort was not consistent at each site visit, and this study therefore consists of a convenience sample. However this is accounted for in analyses as described in the following sections.

**Table 1:** Data on ST frequencies among hosts/sources.

ST	DM	ECH	RBV	RS	WFM	Ticks	MB	ONRv	QC	MR
1	0	0	1	0	7	9	0	0	9	8
3	0	1	0	1	4	14	0	0	11	9
4	0	0	0	0	1	1	0	0	1	1
8	0	1	0	0	0	1	0	0	2	0
9	0	0	0	0	3	3	0	0	2	4
12	0	1	0	0	0	7	0	1	4	3
14	0	3	0	0	0	0	0	0	2	1
16	0	0	0	0	0	4	0	0	3	1
19	0	1	0	0	0	2	0	0	1	2
29	0	1	0	0	0	0	0	1	0	0
32	1	0	0	0	0	0	0	1	0	0
36	0	0	0	0	1	1	0	0	1	1
46	0	0	0	0	0	1	1	0	0	0
59	0	1	0	0	1	2	0	0	2	2
222	0	2	0	0	0	0	0	2	0	0
225	0	0	0	1	0	0	0	1	0	0
228	0	1	0	0	0	0	0	1	0	0
234	0	1	0	0	0	0	0	1	0	0
300	0	1	0	0	0	0	0	1	0	0
302	1	0	0	0	0	0	1	0	0	0
315	0	0	0	0	0	1	0	0	1	0
519	0	0	0	0	0	1	0	0	1	0
532	0	1	0	0	0	0	0	1	0	0
535	0	0	0	0	1	0	0	0	0	1
536	0	0	0	0	1	0	0	0	0	1
537	0	0	0	0	0	1	0	0	0	1
538	0	0	0	0	1	0	0	0	0	1
641	0	1	0	0	0	0	0	1	0	0
643	0	0	0	0	0	1	0	0	1	0
644	0	0	0	0	0	1	0	0	1	0
Total	2	16	1	2	20	50	2	11	42	36

The number of *B*. *burgdorferi* MLST sequence types among 5 host species used in this study: *Peromyscus maniculatus* (DM: Deer mouse), *P*. *leucopus* (WFM: White-footed mouse), *Tamias striatus* (ECH: Eastern chipmunk), *Tamiasciurus hudsonicus* (RS: Red squirrel) and *Myodes gapperi* (RBV: Red-backed vole), and host-seeking ticks (Ticks) sampled in four different regions from Southern Canada: Manitoba (MB), Ontario at Rainy River (ONRv), Quebec (QC) and the Maritimes (MR).

**Fig 1:**
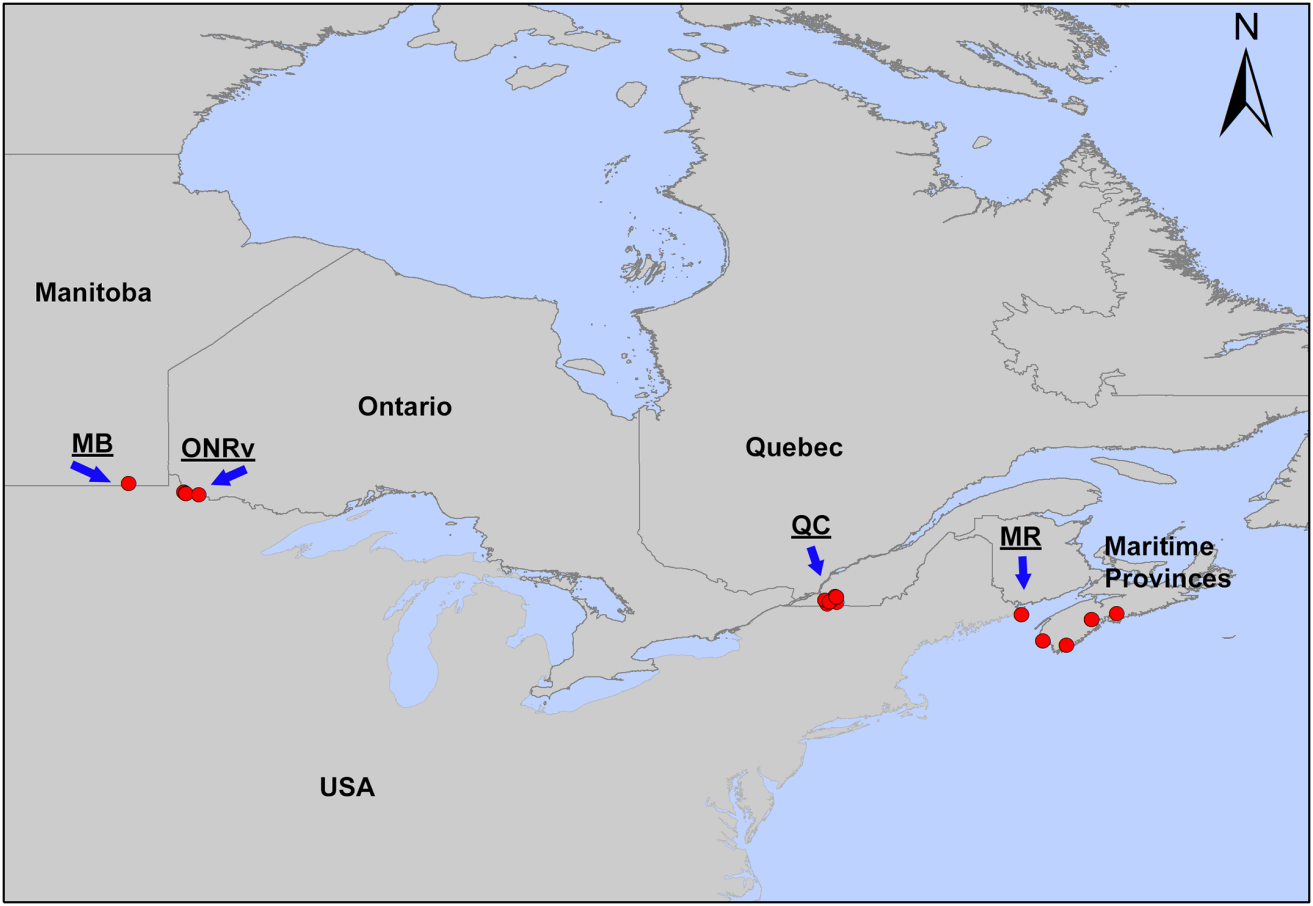
Geographic distribution of the sampling sites in Canada. A map of northern North America showing the names of the main Canadian provinces. Red dots indicate the locations of sample sites in each of the four regions where samples from animal hosts were available which are indicated by the abbreviations used in the text (MB = Manitoba, ONRv = Rainy River Ontario, QC = southern Quebec and MR = the Maritime provinces of Nova Scotia and New Brunswick).

Locations of sites where ticks and/or rodents were collected include those presented in [26] as well as more recently visited sites in northwestern Ontario. To facilitate analyses, study sites that were in close proximity were grouped into six geographic regions as follows: (1) Manitoba (MB; 8 sites), (2) Ontario Rainy River (ONRv; 4 sites), (3) Ontario Long Point (ONLp; 1 site); (4) Ontario East (ONEst; 7 sites), (5) Quebec (QC; 16 sites), and (6) the Maritimes (MR; 14 sites). All samples from these regions were used in phylogenetic analyses, however only questing ticks, without contemporaneously collected rodent host samples, were available from ONLp and ONEst. Data from only four regions (MB, ONRv, QC and MR), where rodent samples and questing ticks were collected contemporaneously, were therefore used in statistical analyses, and the locations of the sites in these regions are shown in Fig 1. The full range of sites in the US and Canada where samples have been collected for phylogenetic analysis (excluding those from ONRv, which are the most recently sampled sites) is shown in Fig 1 of reference [30]. The regions of MB and ONRv combined, and QC and MR comprise regions of emergence of *B*. *burgdorferi* in Canada and we have no reason to believe that the sites from which rodent samples were collected in this study were in any way outliers compared to other sites in these regions in terms of rodent host species (with the caveat that deer mice predominate over white-footed mice in the more western regions and vice versa in the eastern regions) and *B*. *burgdorferi* genotypes.

### Genotyping *B*. *burgdorferi*

For this study, we focused on genotyping by MLST using eight chromosomal housekeeping genes as previously described [29, 41]. However, we also amplified, sequenced and analysed 16S-23S *rrs-rrlA* intergenic spacer (IGS) sequences, as well as the outer surface protein C gene (*ospC*), which have both been used in genotyping of *B*. *burgdorferi* [42, 43]. Any samples that showed evidence of mixed-genotype infections on examination of the housekeeping genes and IGS sequences were excluded [30]. Of the 91 samples analysed for the first time in this study, all had MLST data, 80 had IGS data and 69 had *ospC* data. The difference in the number of samples was due to the lack of available DNA for some samples.

Genotyping using the MLST scheme was conducted as previously described [30, 41]. Fragments of the eight housekeeping genes (clpA, clpX, nifS, pepX, pyrG, recG, rplB and uvrA) were amplified, and the resulting sequences were assigned to existing or new allele numbers (for novel sequences) using the MLST database (http://www.pubmlst.org). The allele combination for each sample was assigned to an existing or to a new sequence type (ST) number (for genotypes with novel alleles or novel allele combinations). All data from this study are available at http://www.pubmlst.org. Clusters of related STs were then identified as follows. MLST STs were ‘classified’ into clonal complexes obtained using eBURST V.3 [44] to reconstruct relationships between *B*. *burgdorferi* STs identified in this study. This clustering method allows clonal complexes to be constructed using different criteria for the relatedness of the STs: single locus variants and double locus variants (SLV and DLV). Each sample was assigned membership to clonal complexes defined using criteria of both SLV and DLV. The confidence in the relationship of the member of each clonal complex was computed by the spanning edge betweenness (SEB) corresponding to the percentage of the equivalent minimum spanning trees (MSTs) between STs of the same clonal complex (i.e. the optimal edge selected by the goeBURST algorithm is the most frequently reproduced edge in the MST forest of the clonal complex), which is expressed as a bootstrap value [45]. An unrooted Bayesian phylogenetic tree of the aligned STs without outgroups was constructed using MrBayes v3.2.1 [46] to support the SEB values, which allowed the posterior probability of each corresponding clonal complex at SLV and DLV to be deduced, and for CCs to be visualised alongside the different clades of *B*. *burgdorferi*. A rooted Bayesian phylogenetic tree of the aligned STs was also generated using MrBayes v3.2.1, in which Markov Chain Monte Carlo samplings were run for 500,000 generations, with trees sampled every 1,000th generation. To define the phylogenetic groups by the eBURST analysis, we used data obtained from 750 samples including all 273 samples from Canada from this and previous studies [26, 30], 477 samples of *B*. *burgdorferi* collected from questing ticks and/or from ticks on hosts in the USA, and 19 samples identified to date only in human patients in the USA. All these data are freely available in the pubmlst.org database. From these samples, 138 unique STs were available for use in the phylogenetic analyses. To support interpretation of the rooted phylogenetic tree in terms of the recent diversification of the different STs, a minimum spanning tree (MST) was constructed using goeBURST. The goeBURST analysis uses the distinct numerical allelic profile of each ST to reconstruct the phylogenetic links between genotypes and to infer an evolutionary descent pattern [47]. The eBURST algorithm within goeBURST defines the primary founder ST in the group of STs as the ST that has the greatest number of single-locus variants within the population of STs [44]. For this analysis the strength of the link between two STs is given as the SEB value (obtained up to triple locus variants: TLV), and the number of locus differences between them.

The intergenic spacer 16S-23S *rrs-rrlA* was amplified from the samples by nested PCR using the outer primers PA Forward (GTATGTTTAGTGAGGGGGGTG position: 2306–2326) and P95 Reverse (GGATCATAGCTCAGGTGGTTAG position: 3334–3313), and the inner primers PB Forward (AGGGGGGTGAAGTCGTAACAAG position: 2318–2339) and P97 Reverse (GTCTGATAAACCTGAGGTCGGA position: 3305–3284) as described in [42]. For comparison of IGS sequences from samples in this study with those from previous studies, we classified our samples according to three previously-used methods. First, IGS sequence type identification numbers were assigned according to the scheme of [42] by comparing the sequences from our samples to reference sequences available in Genbank (accession numbers AY275189 to AY275212). Second, we assigned expected Ribosomal Sequence Types (RSTs) as reported in [48] because this is frequently used to distinguish (in broad terms) genotypes of *B*. *burgdorferi* that have ecological differences and vary in pathogenicity [31, 48]. To do this we assigned ribosomal spacer identification numbers (RSPs) to deduced IGS types according to the method of [49] by comparing our sequences with the relevant reference sequences in Genbank (accession numbers: EF649781 for RSP1, EF649783 for RSP3, EF649784 forRSP4, EF649786 for RSP6, EF649787 for RSP7, EF649789 for RSP9, EF649790for RSP10, and EU477177 to EU477185 for RSP12 to RSP20). Then, again following [25], RST numbers were assigned to the following RSPs [25]: RST1 corresponding to RSP1 and RSP7; RST2 corresponding to RSP3, RSP4 and RSP20; and RST3 corresponding to RSP14, RSP9 and RSP18, RSP10, RSP12 and RSP13, RSP19 (Table A in S1 File). An unrooted Bayesian phylogenetic tree was constructed from the 80 available IGS sequences from the samples investigated here, with 16 reference ribosomal sequence spacers (RSP) and 24 IGS type and subtype sequences downloaded from GenBank, using MrBayes.

The *ospC* gene was also amplified by semi-nested PCR using the outer primers OC6 (+) (AAAGAATACATTAAGTGCGATATT) and 623 (-) (TTAAGGTTTTTTTTGGACTTTCTGC), and the inner primers OC6 (+Fluo) (Fluorescein-AAAGAATACATTAAGTGCGATATT) and 602 (-) (GGGCTTGTAAGCTCTTTAACTG) as reported in Qiu et al. 2002 [43]. The *ospC* major groups were identified by multiple alignment performed in ClustalW2 using default settings [50]. Pairwise alignment was done using reference sequences downloaded from GenBank (accession numbers are EU482041 to EU482051 for *ospC* major groups A to K, EU375832 for *ospC* L, EU482052 and EU482053 for *ospC* M and N, EU482054 and EU482055 for *ospC* T and U, EF592542 for *ospC* B3, EU482056 for *ospC* E3, EF592547 for *ospC* F3, HM047876 for *ospC* X and HM047875 for *ospC* Y). The criteria for assigning *ospC* sequences to *ospC* major groups were those described by [43] *i*.*e*. difference similarity of ≥ 99% to be included in a major group, and a similarity of ≤ 90% to be excluded from a major group.

### Data analyses

#### Diversity of rodents and *B*. *burgdorferi* genotypes among study sites

With unequal sampling effort in different sites and regions, it is difficult to compare the diversity of *B*. *burgdorferi* genotypes and host species (using richness as an index) among different locations. However, development of individual rarefaction curves [51, 52], is a commonly used method for comparing species richness among samples with unequal sampling effort [53]. This approach allows comparison between different communities/locations after each community/location is "rarefied" back to an equal number of sampled specimens [51, 54, 55]. Three analyses were performed using PAST version 2.17c [56]. These included analysis of host species richness by geographic region, and *B*. *burgdorferi* genotype richness by host species and region. For this analysis, *B*. *burgdorferi* genotypes were MLST sequence types (STs) [57]. With the exception of the analysis of host species richness, in these and subsequent analyses we considered that STs in questing ticks as well as STs obtained from hosts (directly from infected host tissues or from one engorged tick collected from the host) as comprising different ‘ecological sources’ of *B*. *burgdorferi* genotypes. In doing so, we recognized that the frequency of STs in questing ticks is the product of the transmission of *B*. *burgdorferi* genotypes from all species of the tick-host community in a particular location (see below).

In order to compare the abundance and the frequency of *B*. *burgdorferi* genotypes in broad terms among the field sites and animal hosts, the observed distributions of these STs among hosts and regions were compared against their expected distributions (*i*.*e*. the mean value of the number of identified STs among the host species and/or geographic regions). This comparison was performed using the chi-squared goodness of fit test where the null hypothesis was that the abundance of genotypes is the same among different host species. The alternative hypothesis is that the STs are non-randomly distributed among hosts suggesting possible associations with host species. The test was conducted with 95% confidence intervals using Fisher’s exact test by the Monte Carlo Estimation algorithm in SAS version V9.4 (SAS Institute Inc., Cary, NC, USA).

#### *Borrelia burgdorferi* genotype-host species associations

Samples used in this analysis were one positive engorged tick or tissue sample per infected host, as well as questing ticks collected in the locations where the small mammal trapping was conducted. The null hypothesis was that the proportion of samples positive for a particular genotype would be the same amongst sources (questing ticks and different species). Questing ticks were included in this analysis as a category because the frequency of different genotypes in each trapping location to which hosts are exposed equals the prevalence in the questing tick population, so it would be expected, in the absence of genotype-host species associations, that frequencies of genotypes in questing ticks and in hosts would be similar.

First, correspondence analysis was performed using SPSS V17 (SPSS Inc., Chicago, US) to explore the relationship between *B*. *burgdorferi* genotypes and host species/source and their geographic locations. The analysis was conducted for different levels of clonal complex inference (*i*.*e*. single locus variant and double locus variant).

The correspondence analysis informed the development of logistic regression models to individually assess associations between genotypes of *B*. *burgdorferi* (determined by clonal complexes, *ospC* major groups, and RSTs) and host species/source. Mixed effects generalized linear models with a logit link function were developed in SAS 9.4, where the fixed effects were host species/source, while the number of sampling visits (as a category rather than as a continuous variable) and geographic region of origin with nested individual site ID numbers were both considered as random effects in the same models. Site ID nested by region was included as a random effect to account for regional and inter-site variations that were not explicitly explored, while the number of visits (as a category) was explored as a random effect as different numbers of visits per site may have been associated with different probabilities of finding genotypes by (for example) reflecting different seasons of sampling. For this purpose, the GLMM (Generalized linear mixed model) with GLIMMIX procedure was performed in SAS version 9.4.

The general models were structured as follows:

$$CCi=\;\beta_{0}+\beta_{1}+\text{(}visit|region/siteID)$$

Where *CC*_*i*_ is the clonal complex/*ospC* or RST type as a binary outcome (*i*.*e*. 0 = absence and 1 = presence); *β*_*0*_ is the estimate of the occurrence of the *CC*_*i*_ when all covariates are equal to zero; *β*_*1*_ represents the fixed effects for the host species/source and the random effects of the number of site visits and the site ID, nested by region, indicated in brackets.

The models were fitted using a non-blocked covariance matrix assumption and parameter estimates were obtained using Restricted Maximum Likelihood to avoid certain deficiencies of the Maximum Likelihood method which does not take into account the loss in degrees of freedom due to the use of the fixed effect estimator [58]. The robust standard error estimator (empirical ‘sandwich’ estimators in the GLIMMIX procedure of SAS) was used to ensure results using small sample sizes were more robust [59]. A backward elimination process was used to group host species that were not significantly different, although questing ticks remained the reference host/source throughout. To further explore the significance of findings, minimal models were compared statistically against intercept models, and analyses were recreated in R version 3.2.3 (The R Foundation for Statistical Computing, Vienna, Austria) using logistic regression to see if statistical significance remained using different model constructions. When significant differences in genotype occurrence amongst hosts were found when including data from questing ticks in statistical models, the analyses were repeated without questing tick data to see if these associations remained significant to provide more robust evidence of host-genotype associations. The level of significance was P < 0.05.

## Results

### Genotyping of *B*. *burgdorferi*

Of the samples collected at the study sites, 437 were analyzed by MLST (for which 273 were successfully sequenced with 34 samples being rejected as having mixed infections), *ospC* (for which 240 were successfully sequenced with 25 samples being rejected as having mixed infections), IGS (for which 258 were successfully sequenced with 30 samples being rejected as having mixed infections). Following removal of multiple samples from hosts there were 91 samples (41 from hosts and 50 from questing ticks) for statistical analyses of host-genotype associations.

**Fig 2:**
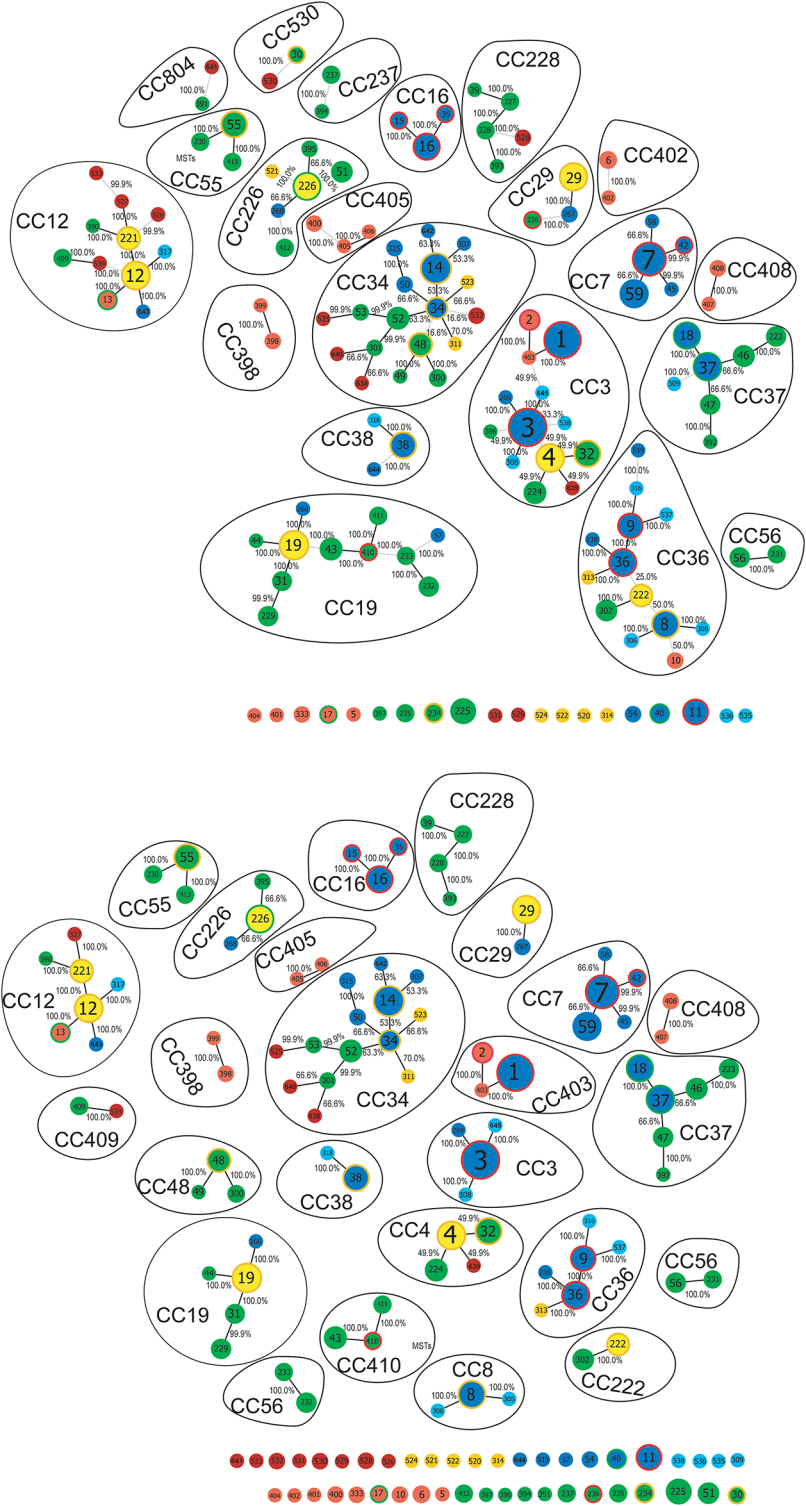
MLST clonal complexes. Clonal complexes and singletons identified by eBURST and goeBURST analyses constructed at SLV (A) and DLV (B) levels. The spanning edge betweenness value (expressed as %) of the optimal edge is reported. STs are color-coded according to their geographic location: blue, STs found in the ‘northeast’ (*i*.*e*. STs found in Northeastern US and also in Quebec, eastern Ontario and the Maritimes); green, STs found in the ‘midwest’ (*i*.*e*. STs found in Midwestern US and also in Manitoba); yellow, STs occurring in both the ‘northeast’ and the ‘midwest’; red, STs found in California; cyan, STs found only in the Maritimes; orange, STs found only at Long Point Ontario; brown, STs found only in Manitoba.

The eBURST and goeBURST analyses, using all 750 samples of the full MLST data set, identified 25 clonal complexes and 44 singletons using the SLV criterion, and 21 clonal complexes and 20 singletons using the DLV criterion. The 273 samples from Canada comprised 61 STs occurring in 18 different clonal complexes with 20 being singletons using SLV criterion, and 15 clonal complexes and 9 singletons using the DLV criterion. Using the SLV criterion, the largest clonal complex was CC34 which contained 9 STs (ST14, ST52, ST301, ST315, ST523, ST525, ST638, ST640, ST642), followed by CC12 which contained 4 STs (ST12, ST221, ST527, ST643), and CC4 and CC36 which both contained 3 STs (ST4, ST32, ST639, and ST9, ST36, ST537 respectively). The rest of the clonal complexes were minor complexes; each minor complex contained two sequence types (i.e. 12 STs and 10 others STs linked at least one ST from USA) (Fig 2).

Using the DLV criterion, CC34 was again the largest clonal complex with 12 STs, followed by CC3, CC12, CC36 each with 7 STs, CC19 with 5 STs, and CC226 and CC228 each with 3 STs. The MSTs statistics (SEB) reported in the goeBURST diagram for edges between STs constituting each clonal complex using SLV (Fig 2A) and DLV (Fig 2B) criteria indicate that within clonal complexes the STs are highly related (*i*.*e*. the frequency with which they formed optimal edges in the MSTs forest tree for each CC was between 99.9% and 100%) and correspond mostly to significant (100% of the posterior probability) clades in the unrooted phylogenetic tree (*e*.*g*. CC19, CC55, CC38) (Fig A in S2 File). However, for certain large clonal complexes including CC34 some of the STs (particularly when using DLV criteria) had MST statistics indicating lower relatedness with other clonal complex members (with frequencies of the optimal edges ranging from 16% to 66.6%) and these STs frequently came from different clades in the phylogenetic tree. An example is CC36, which using the DLV criterion, comprises 3 groups of STs from three distinct (with 94% the posterior probability) clades of the unrooted phylogenetic tree (Fig A in S2 File) that are linked by edges reproduced only in ≤ 50% cases in the MST forest tree (Fig 2).

**Fig 3:**
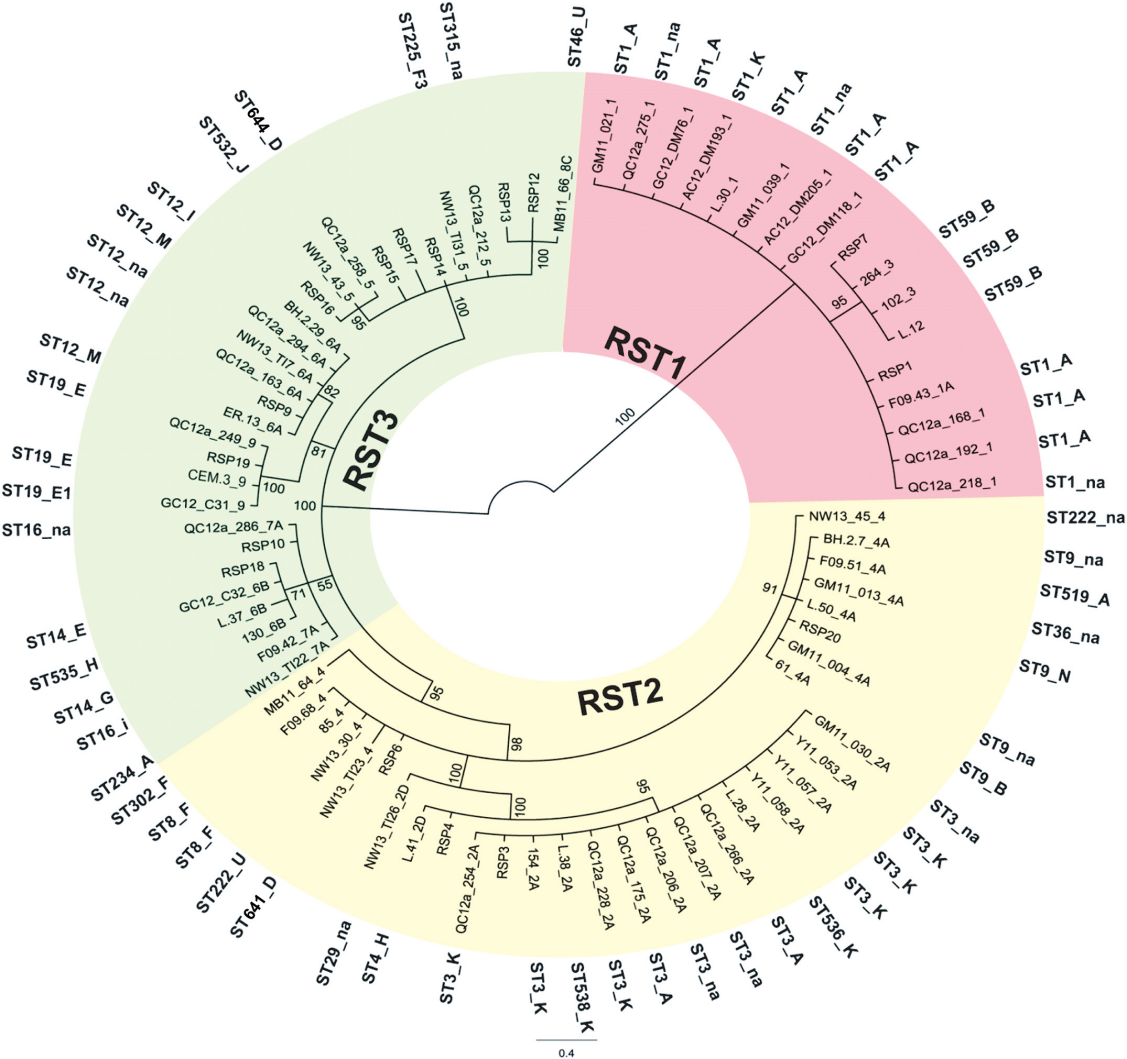
An unrooted Bayesian maximum likelihood tree of ribosomal 16S-23S *rrs-rrlA* IGS spacer sequences. The tree was constructed using the 80 samples for which these sequences were available, as well as reference sequences described in the text. Identification numbers of the samples and the IGS type number are shown on the tree. The correspondence of these sequences to RST types is shown by colour coding (RST1: dark pink; RST2: light pink; RST3: gray). Around the colour-coded areas are the corresponding ST numbers and *ospC* major groups of the samples (na = data not available).

Intergenic spacer sequences were obtained from 80 of the samples used in statistical analyses and comprised 7 IGS types and 5 IGS subtypes (Table B in S1 File). An unrooted phylogenetic tree of these sequences (Fig 3) showed that the sequences and 12 IGS types and subtypes are well clustered into the three distinct ribosomal sequence types, RST1, RST2 and RST3. RST1 contains two IGS types (1 and 3) and one subtype (1A), RST2 contains one IGS type (4) and three subtypes (2A, 2D and 4A), and RST3 was the largest group comprising two IGS types (5 and 9) and four subtypes (6A, 6B, 7A and 8C).

Alleles belonging to 15 major *ospC* groups (including A, B, D, E, E1, F, F3, G, H, I, J, K, M, N and U) were identified among 69 samples used in statistical analyses for which full *ospC* sequence data were available. The most frequent major group found was *ospC* A, which corresponded to 4 STs (ST1, ST3, ST9, ST519), *ospC* K corresponded to ST1, ST3, ST536 and ST538, and other *ospC* major groups were linked to one or two STs (Table B in S1 File). The *ospC* major groups were associated with different RSTs with 80% of *ospC* A being associated with RST1 and 20% associated with RST2, 75% of *ospC* B being associated with RST1 and 25% associated with RST2, while 94% of *ospC* K were associated with RST2 and 6% were associated with RST1. *ospC* major groups E, E1, G, J and M were all associated with RST3 (Table B in S1 File, and Fig 3).

### Diversity of rodents and *B*. *burgdorferi* genotypes among study sites

Details of the origin of the samples and the *B*. *burgdorferi* ST frequencies are shown in Table 1.

**Fig 4:**
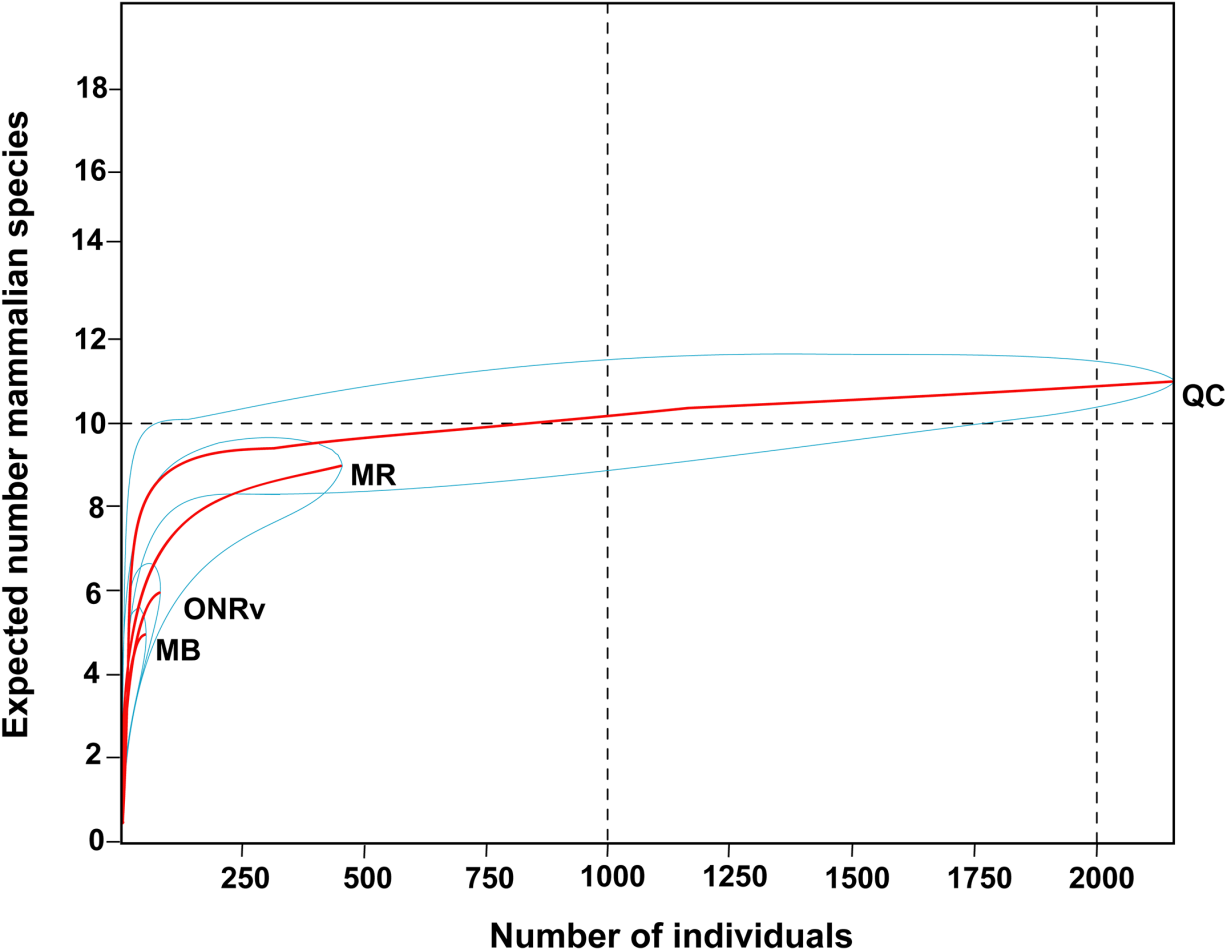
Rarefaction curves of rodent species richness. Comparisons are made among the four geographic zones where rodent trapping was conducted (Manitoba [MB], Ontario Rainy River [ONRv], Quebec [QC] and the Maritimes [MR]) in the richness of rodent communities using rarefaction measurements. Blue lines depict the 95% confidence limits.

The rarefaction curves suggested that mammal species richness was similar among the regions, and that detected specific richness would rise at approximately even rates with increased sampling effort, and plateau at approximately the same sample size, in each region (Fig 4).

**Fig 5:**
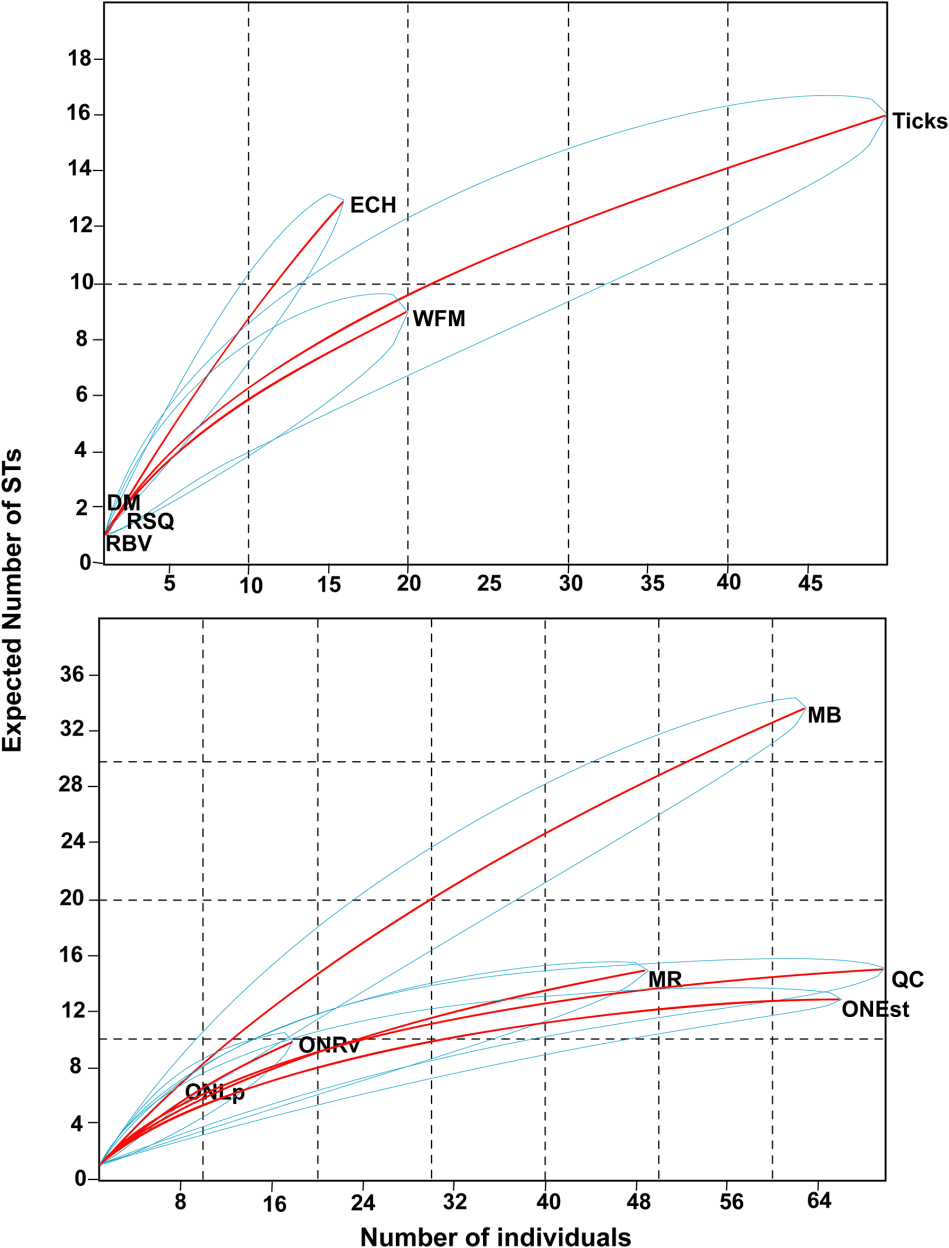
Rarefaction curves of ST richness. Comparisons of the richness of *B*. *burgdorferi* STs using individual rarefaction curves among four different geographic regions (A) and the six ecological sources (B). The geographic regions are Manitoba [MB], Ontario at Rainy River [ONRv], Quebec [QC] and the Maritimes [MR]. The sources are Deer mouse (DM), Eastern chipmunk (ECH), Red-backed vole (RBV), Red squirrel (RSQ), White-footed mouse (WFM), and questing ticks (Ticks). Blue lines depict the 95% confidence limits.

In contrast, among the regions the highest ST richness was in Manitoba (33 STs) followed by Quebec (15 STs) and the Maritimes (15 STs), and the individual rarefaction curves suggested that the detected richness of STs would rise faster by increased sampling effort in Manitoba compared to other regions (Fig 5a).

The richness of STs among host species was highest in the eastern chipmunk with 24% of the total STs (13 STs) followed by white-footed mice with 16% of STs (9 STs), which had similar specific richness of STs to host-seeking ticks. The individual rarefaction curves suggested that the richness of STs would rise faster by increasing sampling effort in eastern chipmunks compared to increased sampling of other rodents or questing ticks (Fig 5b). The chi-square goodness of fit test suggested that *B*. *burgdorferi* genotypes were non-randomly distributed among hosts/sources (χ^2^ = 935.38, df = 6, *P* < 0.001) and among geographic locations (χ^2^ = 78.63, df = 5, *P* < 0.001) (Table 2). Note that the level of statistical significance was adjusted according to Bonferroni correction to *P* < 0.002 (= 0.05/21) for the fixed factor host species/ticks and *P* < 0.003 (= 0.05/15) for the fixed factor geographic location, to account for multiple one-way comparisons. Thus, this analysis suggests that there were significant associations between *Borrelia* genotypes and host species/ticks and between *Borrelia* genotypes and geographic locations. The Mantel-Haenszel chi square test suggests that the relationship between STs and geographic zones was linear (χ^2^ = 8.93, df = 1, *P* = 0.002) supporting the specificity of certain genotype ranges to some locations (Table 2). In both cases the contingency coefficient (Cf) suggests that the genotypes are strongly associated with certain hosts/sources (Cf = 0.80) and geographic locations (Cf = 0.81) (Table 2).

**Table 2:** Chi-square goodness of fit test results.

Host-CCs			
Statistic	DF	Test value	*P*
Chi-Square	6	935.38	< 0.001
Monte Carlo Estimate for the F Exact Test			0.001
Mantel-Haenszel Chi-Square	1	2.69	0.101
Monte Carlo Estimate for the F Exact Test			0.102
Contingency Coefficient		0.80	
Locations-CCs			
Statistic	DF	Test value	*P*
Chi-Square	5	78.63	< 0.001
Monte Carlo Estimate for the F Exact Test			< 0.001
Mantel-Haenszel Chi-Square	1	8.93	0.002
Monte Carlo Estimate for the F Exact Test			0.002
Contingency Coefficient		0.81	

Results for associations among hosts and clonal complexes (CCs), and among locations and CCs are shown.

#### *Borrelia burgdorferi* genotype-host species associations

**Fig 6:**
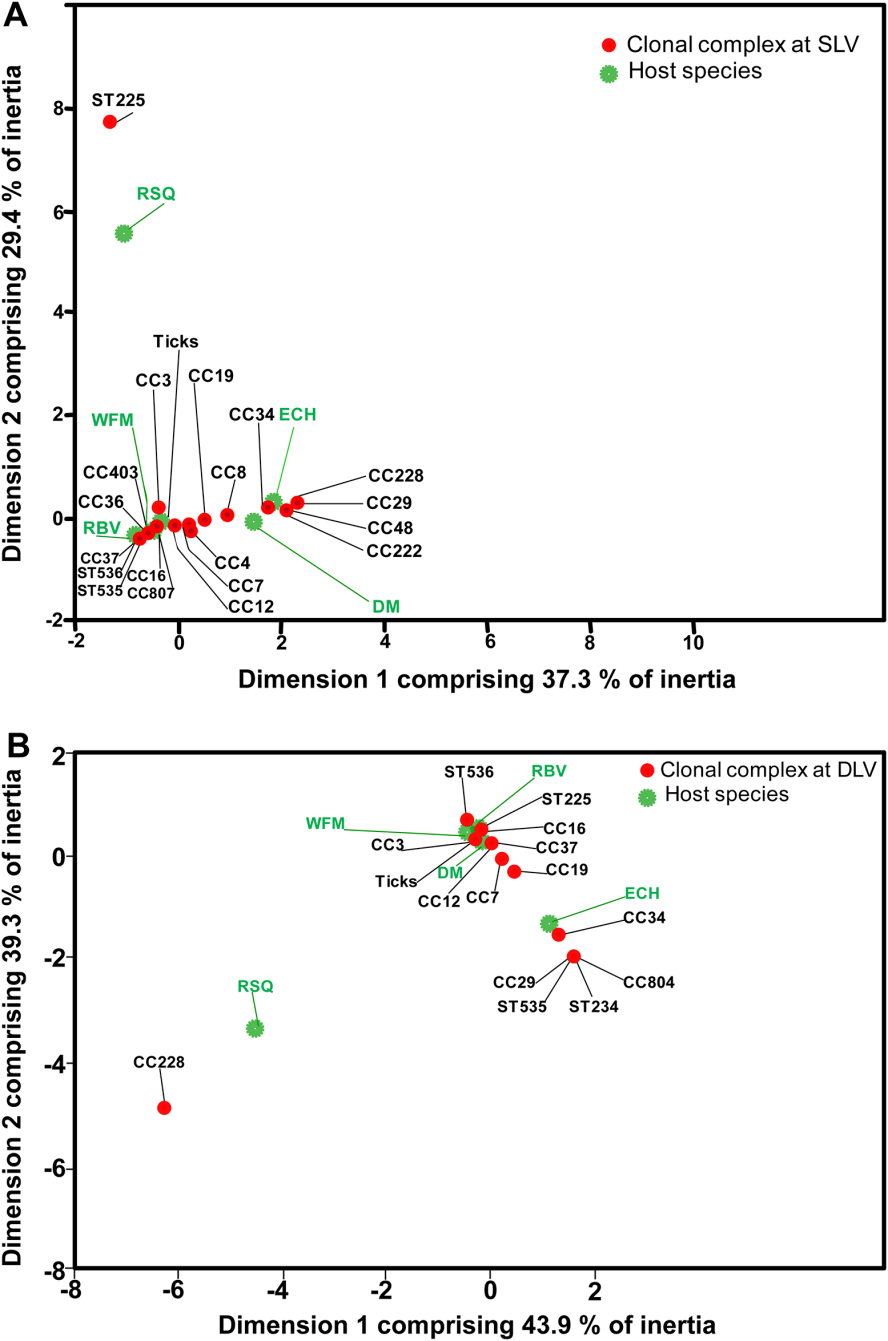
Correspondence analysis results. Correspondence analysis biplot maps are shown for associations among host species/sources and the MLST clonal complexes when these were obtained using SLV (A) and DLV (B) criteria.

For this analysis there were 91 samples comprising 30 STs that, in the goeBURST analysis described above, fell into 15 of the clonal complexes with 8 that were singletons using the SLV criterion (Table C in S1 File), or fell into 11 of the clonal complexes with 5 singletons using the DLV criterion (Table D in S1 File). The correspondence analysis shows evidence of an association between certain host species and clonal complexes suggesting that significant host-genotype associations exist, regardless of whether clonal complexes were formed using SLV criteria (χ^2^ = 158.28, df = 110 and *P* = 0.002) or DLV criteria (χ^2^ = 108.14, df = 75 and *P* = 0.007) (details in Tables E to J in S1 File). The two-dimension solution of the principal component analysis explains 66.7% of the variation when clonal complexes are developed using the SLV criterion (Fig 6A) and 83.2% when clonal complexes were developed using the DLV criterion (Fig 6B) indicating that most of the variation reflected an association between host species and clonal complexes. In both cases, correlation of CC34 and chipmunks is high (Table F and J in S1 File). Correspondence analysis also suggested associations between the white-footed mouse and CC403. The analysis suggested an association between red squirrels and clonal complexes although there were only two individuals of this species in the data set (Table 1, Table F and J in S1 File, Fig 6).

Results of the GLIMMIX model analyses are summarised in Table 3 and detailed in Tables K to T in S1 File. Significant associations were found between chipmunks and STs of CC34 (when constructed by both SLV and DLV criteria) and with RST2 type IGS sequences (IGS4) and *ospC* G. Significant associations were found between white-footed mice and CC403 (which was only present when clonal complexes were constructed with SLV criteria), RST1 type IGS sequences and *ospC* A. Significant associations were also found between deer mice and CC4 (which was only present when clonal complexes were constructed with SLV criteria), and with *ospC* H. In each case the prevalence of these STs and IGS and *ospC* sequences were significantly different from the prevalence in questing ticks and in other host species (Tables K to R in S1 File). Also in each case the significance of the minimal models was supported by being significantly different from the intercept-only model (Table T in S1 File). When the minimal models were reconstructed in R software, all remained significant (with and without questing tick data: Tables U and V in S1 File) except for the associations between white-footed mice and CC403 and between white-footed mice and RST1 type sequences, which were marginally non-significant (P = 0.097 and 0.056 respectively) in models with questing tick data included.

**Table 3:** Significant associations of host species with different genotypes of *B*. *burgdorferi*.

Factor	Estimate	Standard Error	DF	t Value	P > |t|
CC34S*					
Intercept	0.01298	0.07381	22	0.18	0.862
ECH	0.3207	0.09097	66	3.53	0.001
Other host spp.	-0.0146	0.07325	66	-0.20	0.843
CC34D*					
Intercept	0.01951	0.07786	22	0.25	0.804
ECH	0.4083	0.09428	66	4.33	<0.001
Other host spp.	-0.0287	0.07564	66	-0.38	0.705
CC403					
Intercept	0.1800	0.05411	88	3.33	0.001
WFM	0.3500	0.08555	88	4.09	<0.001
Other host spp.	0.04762	0.08349	88	0.57	0.570
CC4					
Intercept	0.06157	0.06311	22	0.98	0.340
DM	0.4662	0.1296	66	3.60	0.001
Other host spp.	-0.0279	0.05841	66	-0.48	0.633
RST 1 type IGS sequences*					
Intercept	0.1905	0.06084	77	3.13	0.002
DM	0.3500	0.08817	77	3.97	<0.001
Other host spp.	0.05556	0.09293	77	0.60	0.552
RST 2 type IGS sequences (IGS4)					
Intercept	0.02381	0.03814	77	0.62	0.534
ECH	0.2839	0.07846	77	3.62	<0.001
Other host spp.	0.01619	0.06244	77	0.26	0.796
RST 2 type IGS sequences (IGS2D)					
Intercept	0.02381	0.03226	77	0.74	0.463
DM	0.4762	0.1513	77	3.15	0.002
Other host spp.	0.03175	0.04748	77	0.67	0.506
*ospC* G**					
Intercept	-0.00216	0.04874	32	-0.04	0.965
ECH	0.2471	0.07165	34	3.45	0.001
Other host spp.	-0.00637	0.05550	34	-0.11	0.909
*ospC* A					
Intercept	0.2632	0.06698	66	3.93	<0.001
WFM	0.3529	0.1001	66	3.52	0.001
Other host spp.	-0.2632	0.1291	66	-2.04	0.050
*ospC* H					
Intercept	0.02632	0.03647	66	0.72	0.473
DM	0.4737	0.1631	66	2.90	0.005
Other host spp.	0.04265	0.05543	66	0.77	0.444

Throughout the prevalence of the genotypes in questing ticks was the reference value for analyses. Full data are presented in Tables K to S in S1 File. Host species abbreviations are: DM = deer mouse, ECH = eastern chipmunk and WFM = white-footed mouse. * indicates that the random effect of site ID nested by region was significant and included in the model while ** indicates that the random effect of the number of site visits was significant and included in the model.

#### Phylogenetic analysis of the host-genotype associations

**Fig 7:**
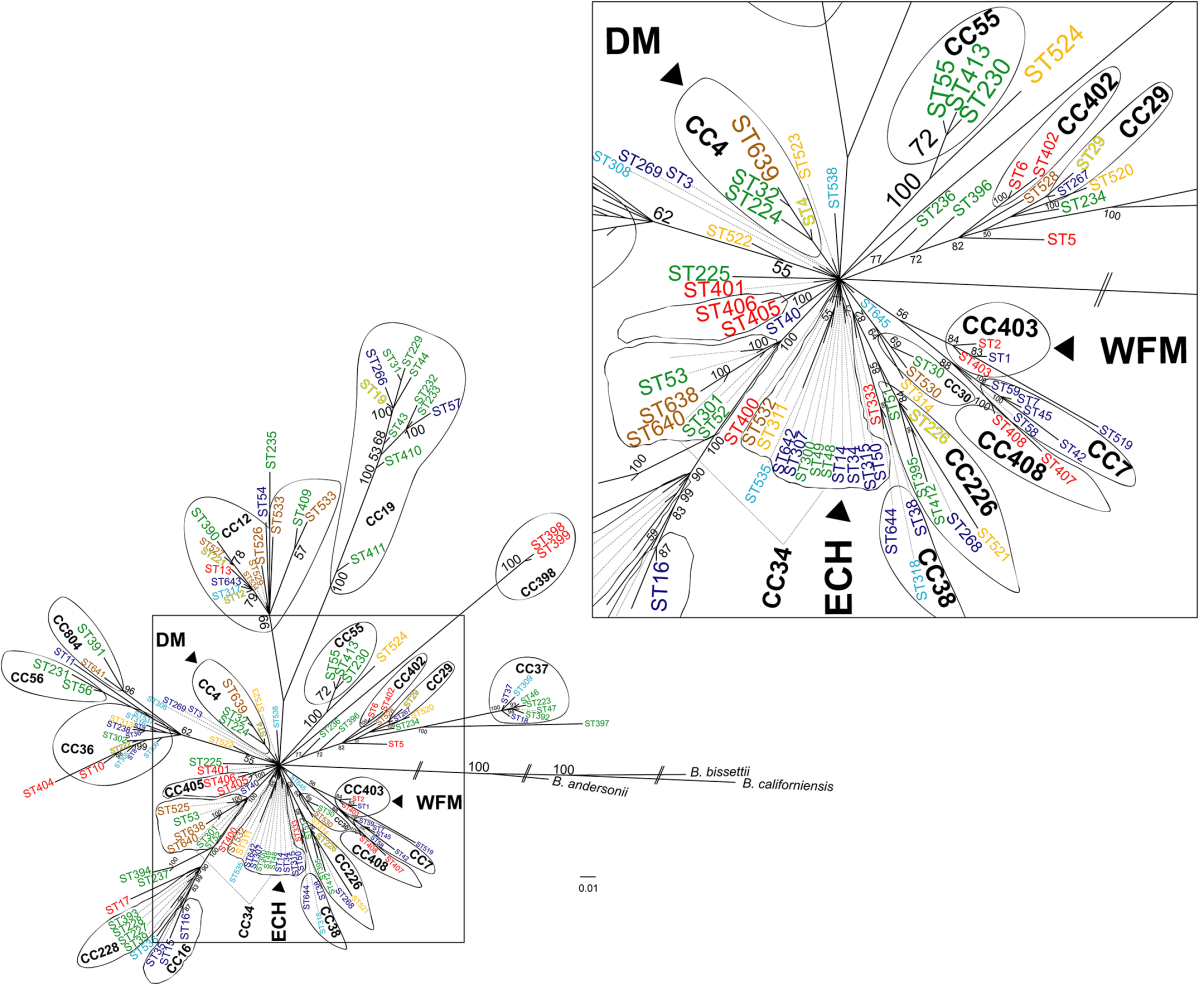
A rooted Bayesian phylogenetic tree of MLST STs. STs are color-coded according to their geographic location: cyan: Maritime Provinces, orange: Long Point Ontario, brown: Manitoba, blue: Northeastern USA, eastern Ontario and southwestern Quebec, green: Midwest USA, yellow: STs found in both northeastern and Midwestern USA, and red: California. Posterior probabilities are shown beside nodes. The scale bar corresponds to the number of substitutions per unit branch length. STs of clades that belong to distinct clonal complexes are encircled and numbered as in Fig 2. ECH, WFM and DM indicate STs of clonal complexes associated with chipmunks, white-footed mice and deer mice respectively. Outgroups are *B*. *bissettii*, *B*. *andersonii* and *B*. *californiensis*. An enlargement of the central part of the tree is shown to the right.

**Fig 8:**
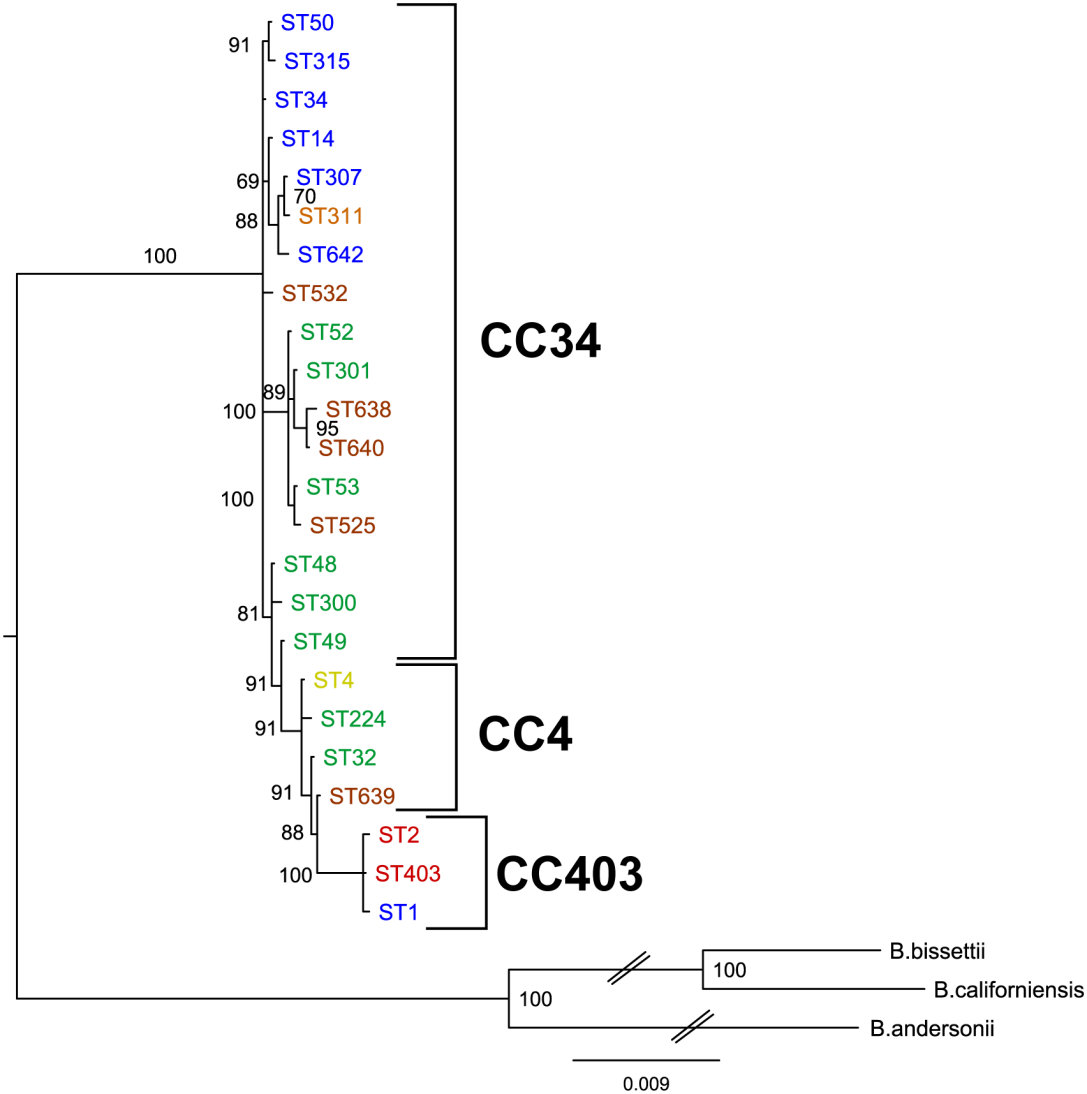
The phylogenetic relationship of STs of clonal complexes associated with rodents. This shows a phylogenetic tree constructed using the same method and the same outgroups as the tree in Fig 7, but with only the STs of the rodent-associated clonal complexes CC34, CC4 and CC403.

The rooted Bayesian phylogenetic tree with outgroups (Figs 7 and 8) shows that many clonal complexes (e.g. CC37, CC403, CC226, CC16) are correlated with clades. However, certain clonal complexes such as CC34 do not form clear clades. For CC34 some STs (ST52, ST53, ST301, ST525, ST638, ST640) form a clear clade with 100% posterior probability, while the rest (*e*.*g*. ST14, ST48, ST300, ST532) group at the base of the tree (Fig 7). For clarity, the relationships of STs among CCs 34, 4 and 403 in a phylogenetic tree constructed using only the STs that are members of these CCs are shown in Fig 8.

The association of eastern chipmunks with CC34 was due to associations with ST14, ST300 and ST532, which are linked in one part of CC34 (Fig 2). There was evidence of associations between deer mice and white-footed mice respectively with CC4 and CC403, STs of which form (on the basis of branch length) more recently evolved clades of the phylogenetic tree (Fig 8). The MST developed using goeBURST shows the optimal parent-descendent linkage among the STs (Fig B in S2 File). For the SLV, DLV, and TLV criteria, ST34 is always predicted as the founder ST with a maximum bootstrap value of 83% obtained at SLV. This means that while the SEB statistics between ST34 (the overall founder and founder of CC34) and ST3 (the founder of CC3), and between ST3 and ST4 (the founder of CC4) and ST1 are low (possibly suggesting horizontal gene transfer: [445]), ST34 may be ancestral to ST3 of CC3, which in turn is most likely to be ancestral to ST1 of CC403 and ST4 of CC4.

## Discussion

To our knowledge, *B*. *burgdorferi* remains a generalist pathogen which can survive in and be transmitted from many host species [4]. In this study we explored possible statistical associations between genotypes of *B*. *burgdorferi* with different host species. The samples available to us came from multiple studies and we used a number of techniques to carefully control for sampling effects. First, we used rarefaction curves to explore whether sites likely differed in the ranges of host species and *B*. *burgdorferi* genotypes, and whether the range of *B*. *burgdorferi* genotypes differed among host species/sources of *B*. *burgdorferi* DNA. This analysis suggested that sites sampled in the different geographic regions had similar host species richness, but *B*. *burgdorferi* genotype richness was higher in the Manitoba sites compared to sites in other regions. This result is consistent with previous studies in the USA that suggest that *B*. *burgdorferi* genotype richness is higher in the upper Midwest (*i*.*e*. immediately south of Manitoba) than in the northeast (*i*.*e*. immediately south of eastern Ontario, Quebec and the Maritimes) [14]. Greater seasonal synchrony of activity of larval and nymphal *I*. *scapularis* ticks may explain these geographic differences in *B*. *burgdorferi* genotype richness. Greater seasonal asynchrony of immature tick activity in the US northeast, with nymphs infecting hosts when they are active in spring to early summer and larvae acquiring infections possibly some months later in late summer, has been associated with higher frequencies of genotypes that have putatively long-lived infections in reservoir hosts [60]. This is consistent with the idea that seasonal asynchrony selects for such genotypes [4, 61]. It has been hypothesised that greater persistence of transmissible infections in the host requires greater adaptation to particular host species [4, 61], so seasonal synchrony of immature ticks in the US Midwest may permit transmission of a wider diversity of less host-adapted genotypes than in the northeast. The rarefaction curves suggest some differences in richness of genotypes among host species, which was supported by the abundance analysis conducted by the goodness of fit statistic. In particular genotype richness was highest in chipmunks and this could be due to the relatively long life (up to three years: [62]) of this species compared to the other dominant rodent species such as white-footed mice which rarely survive for a year [63]. The longer a host survives, the more *B*. *burgdorferi*-infected tick bites it will receive so, with the often persistent nature of *B*. *burgdorferi* infections, longer lived species would be expected to exhibit a wider range of genotypes on the assumption that genotypes are not absolute host specialists. Furthermore, chipmunks may carry higher numbers of nymphal ticks than mice, thus further enhancing their capacity to be infected with a higher diversity of genotypes [14].

The possibility of associations among host species and *B*. *burgdorferi* genotypes was initially explored by correspondence analysis, which suggested that there were non-random associations of genotypes among host species. This was then further explored by generalised linear models which demonstrated, in separate analyses, the following associations: i) one clonal complex (CC34 using both SLV and DLV criteria), RST type 2 IGS sequences (particularly IGS type 4) and *ospC* major group G with chipmunks; ii) one clonal complex (CC4) and *ospC* major group H with deer mice and iii) one clonal complex (CC403), RST type 1 sequences and *ospC* major group A with white-footed mice. The analyses accounted for geographic location, uneven sampling among sites, and small sample sizes. The associations of *Borrelia* genotypes with chipmunks were robust throughout the study, and chipmunk samples were available from nearly all geographic regions (Manitoba, northwestern Ontario, southeastern Ontario-southern Quebec and the Maritimes). The mouse-genotype associations were based on limited sample sizes, and samples from deer mice and white-footed mice were limited in their geographic distribution (deer mouse samples from Manitoba and northwestern Ontario, and white-footed mouse samples from Quebec and the Maritimes). Two of the associations with white-footed mice (CC403 and RST1 sequences) were marginally non-significant when models were run in R when questing tick data were included. Furthermore, although the great majority of our carried single (or single dominant) genotype infections, information from mixed genotype infections was excluded. It would be expected that if hosts are bitten by ticks with mixed-genotype infections then, unless there is some form of selection in the host (other than the “first arriving genotype taking all” principle [64]) frequencies of genotypes in mixed-genotype infections and single-genotype infections should be similar although more research on mixed-genotype infections is needed. Therefore, further explorations of these host-genotype relationships are warranted. Nevertheless, some associations (*e*.*g*. associations of CC403 with white-footed mice) were consistent for logistic regression and correspondence analysis, and associations of *ospC* A sequences with white-footed mice and *ospC* G with chipmunks are consistent with other studies [24]. It was not surprising that all three genotyping methods (MLST, *ospC* and RST types) found associations between host species and *Borrelia* genotypes. In previous studies of the genetic diversity of *B*. *burgdorferi* using the same typing methods, it has been found that *ospC* major groups are often associated with the same STs [41] and there is an overall association between *ospC* major groups and *rrs-rrlA* IGS RSTs (e.g. *ospC* A and B with RST1: [65]), due to relatively high levels of linkage disequilibrium. Here, there was partial evidence of linkage disequilibrium with the STs, IGS types and *ospC* major groups associated with the same host species being found in the same samples in many cases, but not in all (see Fig 4). Samples with ST1 frequently carried *ospC* major group A and IGS sequences were of type 1, all of which were associated with white-footed mice, but some samples with ST1 and IGS type 1 had *ospC* sequences of major group K. Samples with ST14 (of CC34) also carried *ospC* major group G, but not IGS group 4, when all were associated with chipmunks. The one sample from deer mice with all three sequences was ST4, IGS type 2D and carried *ospC* major group H.

There was no evidence in our data for the type of near-complete host specialisation of clonal complexes that is seen with European *Borrelia* genospecies, which involves a well-documented mechanism of genospecies-specific sensitivity to the alternative pathway of complement of different host species [15]. Clear one-to-one host species *ospC* group associations would not be expected as this is not seen in European genospecies [66]. While the prevalence of STs of CC34 (using DLV criteria) in chipmunk samples was 31% (5/16) compared to 2% (1/50) in questing ticks, 69% of genotypes in chipmunk samples were of different clonal complexes. Similarly, the prevalence of STs of CC403 was 35% (7/20) in white-footed mouse samples and 18% (9/50) in questing ticks so 65% of STs in white-footed mouse samples were of different clonal complexes. Associations of genotypes of North American *B*. *burgdorferi* are most likely due to characteristics of the genotypes of a more subtle nature that enhance the likelihood of finding them in, or being transmitted from, a particular host species. These may include particular susceptibility of the host species to the genotype, less pathogenic effects of the genotype in the host species (although pathogenicity in wild hosts is not a common feature [67]), longer persistence of infection and infectivity (*i*.*e*. transmissibility to feeding ticks) of the genotype in the host species [25], more efficient transmission of the genotype from the host species to feeding ticks, and possibly support of co-feeding transmission of ticks in addition to transmission from systemic host infections as previously reviewed [4, 61]. Further research is needed to elucidate the mechanisms that underlie these observed associations.

Hanincova et al [14] made comparisons as to the power of MLST typing versus *ospC* alleles to predict pathogenicity of *B*. *burgdorferi* genotypes, but here we do not make any comparisons, and simply identify that host-genotype associations were detected using the range of different methods for typing genotypes that have been applied to study the genetic diversity of *B*. *burgdorferi* [24, 41, 42, 43, 65, 68]. However, the concatenated housekeeping gene sequences used for MLST typing, which show stabilising selection and neutral variation, do provide more robust data for creating phylogenetic trees than *ospC* (which is under balancing selection) and perhaps IGS (which is not thought to be subject to selection: [1]). The rooted MLST phylogenetic tree suggested that STs associated with mice occurred in clades that (on the basis of branch length supported by the minimum spanning tree: Fig B in S2 File) may have evolved more recently than STs associated with chipmunks (Figs 7 and 8). We hypothesise that chipmunk-associated stains are more closely related to ancestral genotypes from which currently circulating chipmunk and mouse associated genotypes have evolved. This hypothesis may also be supported by several pieces of information on the role of chipmunks in transmission of *B*. *burgdorferi* and their current and past geographic distribution.

First, chipmunks can have an important role in the enzootic transmission cycle of *B*. *burgdorferi*; being important hosts for immature ticks, efficiently transmitting the bacterium to ticks, and often being considered as being second to mice in importance as reservoir hosts only because of their lower relative density [69–75]. Therefore, it is possible that chipmunks could maintain transmission cycles of *B*. *burgdorferi* in the absence of mice, particularly for genotypes that may be more adapted to, and transmissible from them. Second, the hypothesis of adaptation to contemporary host species may be supported by the phylogeography of these small mammals. Several comparative studies have suggested that eastern chipmunk, white-footed mice and deer mice had a similar evolutionary history in northeastern and Midwestern North America since the retreat of the ice after the last glacial maximum approximately 20,000 years ago [76, 77]. However, evidence from fossil data [76, 78], suggests that eastern chipmunks were among the rare small mammal species to have survived and persisted in multiple refugia in northern locations during the last glacial period, and experienced a southward expansion towards the central USA when the ice retreated. Therefore, it is possible that populations of genotypes of *B*. *burgdorferi* were maintained by eastern chipmunks (with potentially host specific nidicolous ticks) in these refugia, and expanded when the climate became more favourable in the late Pleistocene for expansions and co-occurrence of tick vectors, *B*. *burgdorferi* populations, as well as other host species such as *Peromyscus* spp. mice [79–81]. Recent studies support the climate-sensitive nature of *P*. *leucopus* distributions [82].

In this study we have provided evidence of host association of genotypes of *B*. *burgdorferi*, and that these associations occur with genotypes that cluster phylogenetically. These findings support the view that the MLST-defined tree topology reflects both demographic processes (population expansions and contractions) and also associations of host species with *Borrelia* genotypes. Host adaptation may have been the driver for the differences in pathogenicity of North American *B*. *burgdorferi* genotypes that are also reflected by the phylogeny and evolutionary history of this bacterium [14]. Further research is needed to better describe the extent and strength of host-genotype associations, to reveal how these occur mechanistically, and to predict their consequences for human health.

## Supporting Information

S1 File Supplementary Tables. Data and statistical analyses tables. Tables of the raw data used for the first time in the study, and of statistical analysis results.
(DOCX)

S2 File Supplementary Figures. These comprise an unrooted Bayesian phylogenetic tree and a minimum spanning tree of the MLST STs.
(DOCX)
