# Supplementary material for: Evidence for Host-Genotype Associations of Borrelia burgdorferi Sensu Stricto
Source: PLoS One. 2016 Feb 22;11(2):e0149345. doi: 10.1371/journal.pone.0149345 (PMC4763156; doi:10.1371/journal.pone.0149345)
Supplement: S1 File — Tables of the raw data used for the first time in the study, and of statistical analysis results. (DOCX) [file pone.0149345.s001.docx]

Table A. The *rrs-rrlA* reference sequences from the study typed using the different methods in this study to identify IGS types, subtypes and RSTs (ribosomal sequence types). The level of similarity of IGS and RSP performed using multiple alignments is shown.

| IGS accession No | IGS type ID*^a^* | IGS subtypeID*^a^* | IGS type ID*^b^* | RST ID*^c^* | level of similarity |
| --- | --- | --- | --- | --- | --- |
| AY275189 | 1 | 1A | RSP1 | 1 | 100% |
| AY275190 | 1 | 1A | RSP1 | 1 | 99.88% |
| AY275191 | 2 | 2A | RSP3 | 2 | 100% |
| AY275192 | 2 | 2A | RSP3 | 2 | 99.63% |
| AY275193 | 2 | 2A | RSP3 | 2 | 99.75% |
| AY275194 | 2 | 2D | RSP4 | 2 | 100% |
| AY275195 | 3 | 3A | RSP7 | 1 | 100% |
| AY275196 | 3 | 3A | RSP7 | 1 | 99.88% |
| AY275197 | 3 | 3A | RSP7 | 1 | 99.88% |
| AY275198 | 3 | 3A | RSP7 | 1 | 99.88% |
| AY275199 | 4 | 4A | RSP20 | 2 | 100% |
| AY275200 | 4 | 4A | RSP20 | 2 | 99.75% |
| AY275201 | 5 | NI*^d^* | RSP14 | 3 | 100% |
| AY275202 | 6 | 6A | RSP9 | 3 | 100% |
| AY275203 | 6 | 6B | RSP18 | 3 | 100% |
| AY275204 | 6 | 6A | RSP9 | 3 | 99.88% |
| AY275205 | 7 | 7A | RSP10 | 3 | 100% |
| AY275206 | 7 | 7A | RSP10 | 3 | 99.88% |
| AY275207 | 8 | 8A | RSP12 | 3 | 100% |
| AY275208 | 8 | 8C | RSP13 | 3 | 99.88% |
| AY275209 | 8 | 8C | RSP13 | 3 | 100% |
| AY275210 | 8 | 8A | RSP12 | 3 | 99.75% |
| AY275211 | 9 | NI | RSP19 | 3 | 99.75% |
| AY275212 | 5 | NI | RSP14 | 3 | 99.88% |

*^a^rrs-rrlA*typed according to Bunikis et *al.* (2004).

*^b^rrs-rrlA*typed according to Hanincova et *al.* (2008).

*^c^rrs-rrlA*typed according to Liveris et *al.* (1995).

*^d^rrs-rrlA*typed according to Bunikis et *al.*(2004).

Table B. The dataset of 91 samples collected in localities in southern Canada and used in this study. The corresponding genotypes identified using the three typing methods (MLST, IGS, *ospC*) are shown. In this and subsequent tables DM = deer mouse, ECH = eastern chipmunk RBV = red-backed vole, RS = red squirrel and WFM = white-footed mouse. Na = sequence data not available.

| NMLID | ST | ospC | IGS | RSP | RST | DNA source | Tick source | Region | Lat | Lon | Year |
| --- | --- | --- | --- | --- | --- | --- | --- | --- | --- | --- | --- |
| MB11-64 | 302 | F | 4 | 6 | 2 | Tick N | DM | MB | 49.05 | -96.48 | 2011 |
| MB11-66 | 46 | U | 8C | 13 | 3 | Tick F | Drag | MB | 49.05 | -96.48 | 2011 |
| NW13-TI5 | 228 | na | na | na | na | Tick M | ECH | ONRv | 48.72 | -94.59 | 2013 |
| NW13-TI7 | 12 | na | 6A | 9 | 3 | Tick M | ECH | ONRv | 48.72 | -94.59 | 2013 |
| NW13-TI22 | 234 | na | 7A | 10 | 3 | Tick M | ECH | ONRv | 48.72 | -94.59 | 2013 |
| NW13-TI23 | 804 | D | 4 | 6 | 2 | Tick M | ECH | ONRv | 48.77 | -94.66 | 2013 |
| NW13-TI26 | 29 | na | 2D | 4 | 2 | Tick M | ECH | ONRv | 48.72 | -94.59 | 2013 |
| NW13-TI31 | 225 | F3 | 5 | 14 | 3 | Tick M | RS | ONRv | 48.72 | -94.59 | 2013 |
| NW13-30 | 222 | U | 4 | 6 | 2 | Heart | ECH | ONRv | 48.72 | -94.59 | 2013 |
| NW13-36 | 300 | na | na | na | na | Heart | ECH | ONRv | 48.68 | -94.16 | 2013 |
| NW13-43 | 532 | J | 5 | 16 | 3 | Heart | ECH | ONRv | 48.73 | -94.61 | 2013 |
| NW13-45 | 222 | na | 4 | 6 | 2 | Heart | ECH | ONRv | 48.72 | -94.59 | 2013 |
| NW13-54 | 32 | H | 2D | 4 | 2 | Heart | DM | ONRv | 48.68 | -94.16 | 2013 |
| F09.51 | 519 | A | 4A | 20 | 2 | Tick F | Drag | QC | 45.31 | -73.01 | 2009 |
| F09.43 | 1 | A | 1A | 1 | 1 | Tick M | Drag | QC | 45.31 | -73.01 | 2009 |
| F09.29 | 16 | I | 7A | 10 | 3 | Tick F | Drag | QC | 45.31 | -73.01 | 2009 |
| F09.42 | 16 | I | 7A | 10 | 3 | Tick M | Drag | QC | 45.31 | -73.01 | 2009 |
| F09.68 | 8 | F | 4 | 6 | 2 | Tick M | Drag | QC | 45.31 | -73.01 | 2009 |
| 812 | 9 | A | 4A | 20 | 2 | Tick F | Drag | QC | 45.31 | -73.01 | 2008 |
| 61 | 9 | B | 4A | 20 | 2 | Tick N | WFM | QC | 45.10 | -72.97 | 2008 |
| 130 | 14 | G | 6B | 18 | 3 | Tick N | ECH | QC | 45.06 | -73.28 | 2008 |
| 816 | 1 | A | 1A | 1 | 1 | Tick F | Drag | QC | 45.31 | -73.01 | 2008 |
| 35 | 3 | K | 2A | 3 | 2 | Tick N | WFM | QC | 45.18 | -73.35 | 2008 |
| 225 | 3 | K | 2A | 3 | 2 | Tick M | Drag | QC | 45.18 | -73.35 | 2008 |
| 42 | 14 | G | na | na | na | Tick N | ECH | QC | 45.18 | -73.35 | 2008 |
| 154 | 3 | K | 2A | 3 | 2 | Tick N | RS | QC | 45.18 | -73.35 | 2008 |
| 85 | 8 | F | 4 | 6 | 2 | Tick N | ECH | QC | 45.12 | -73.21 | 2008 |
| 620 | 1 | A | 1A | 1 | 1 | Tick L | WFM | QC | 45.31 | -73.01 | 2008 |
| 264 | 59 | B | 3 | 7 | 1 | Tick F | Drag | QC | 45.12 | -73.21 | 2008 |
| 102 | 59 | B | 3 | 7 | 1 | Tick L | ECH | QC | 45.12 | -73.21 | 2008 |
| QC12a-154 | 4 | H | 2D | 4 | 2 | Tick N | Drag | QC | 45.28 | -72.98 | 2012 |
| QC12a-163 | 12 | na | 6A | 9 | 3 | Tick N | Drag | QC | 45.28 | -72.98 | 2012 |
| QC12a-168 | 1 | A | 1 | 1 | 1 | Tick N | Drag | QC | 45.28 | -72.98 | 2012 |
| QC12a-172 | 1 | A | 1 | 1 | 1 | Tick N | Drag | QC | 45.28 | -72.98 | 2012 |
| QC12a-175 | 3 | A | 2A | 3 | 2 | Tick N | Drag | QC | 45.28 | -72.98 | 2012 |
| QC12a-176 | 1 | A | 1 | 1 | 1 | Tick N | Drag | QC | 45.28 | -72.98 | 2012 |
| QC12a-192 | 1 | A | 1 | 1 | 1 | Tick N | Drag | QC | 45.28 | -72.98 | 2012 |
| QC12a-198 | 12 | na | na | na | na | Tick N | Drag | QC | 45.28 | -72.98 | 2012 |
| QC12a-206 | 3 | na | 2A | 3 | 2 | Tick N | Drag | QC | 45.28 | -72.98 | 2012 |
| QC12a-207 | 3 | na | 2A | 3 | 2 | Tick N | Drag | QC | 45.28 | -72.98 | 2012 |
| QC12a-212 | 315 | na | 5 | 14 | 3 | Tick N | Drag | QC | 45.28 | -72.98 | 2012 |
| QC12a-218 | 1 | na | 1 | 1 | 1 | Tick N | Drag | QC | 45.28 | -72.98 | 2012 |
| QC12a-222 | 806 | na | 6A | 9 | 3 | Tick N | Drag | QC | 45.28 | -72.98 | 2012 |
| QC12a-228 | 3 | k | 2A | 3 | 2 | Tick N | Drag | QC | 45.28 | -72.98 | 2012 |
| QC12a-249 | 19 | E | 9 | 19 | 3 | Tick N | Drag | QC | 45.28 | -72.98 | 2012 |
| QC12a-251 | 3 | k | 2A | 3 | 2 | Tick N | Drag | QC | 45.28 | -72.98 | 2012 |
| QC12a-254 | 3 | k | 2A | 3 | 2 | Tick N | Drag | QC | 45.28 | -72.98 | 2012 |
| QC12a-258 | 807 | D | 5 | 16 | 3 | Tick N | Drag | QC | 45.28 | -72.98 | 2012 |
| QC12a-266 | 3 | K | 2A | 3 | 2 | Tick N | Drag | QC | 45.28 | -72.98 | 2012 |
| QC12a-275 | 1 | na | 1 | 1 | 1 | Tick N | Drag | QC | 45.28 | -72.98 | 2012 |
| QC12a-280 | 36 | na | na | na | na | Tick N | Drag | QC | 45.28 | -72.98 | 2012 |
| QC12a-285 | 12 | na | 6A | 9 | 3 | Tick N | Drag | QC | 45.28 | -72.98 | 2012 |
| QC12a-286 | 16 | na | 7A | 10 | 3 | Tick N | Drag | QC | 45.28 | -72.98 | 2012 |
| QC12a-293 | 3 | k | 2A | 3 | 2 | Tick N | Drag | QC | 45.28 | -72.98 | 2012 |
| QC12a-294 | 12 | M | 6A | 9 | 3 | Tick N | Drag | QC | 45.28 | -72.98 | 2012 |
| BH.2.7 | 9 | na | 4A | 20 | 2 | Tick F | Drag | MR | 44.37 | -64.27 | 2006 |
| L.37 | 535 | H | 6B | 18 | 3 | Tick L | WFM | MR | 44.37 | -64.27 | 2006 |
| L.50 | 9 | N | 4A | 20 | 2 | Tick L | WFM | MR | 44.53 | -64.50 | 2006 |
| BH.2.66 | 9 | N | na | na | na | Tick M | Drag | MR | 44.37 | -64.27 | 2006 |
| L.28 | 536 | K | 2A | 3 | 2 | Tick L | WFM | MR | 44.53 | -64.50 | 2006 |
| BH.2.59 | 3 | K | na | na | na | Tick M | Drag | MR | 44.37 | -64.27 | 2006 |
| BH.2.55 | 1 | A | na | na | na | Tick M | Drag | MR | 44.37 | -64.27 | 2006 |
| BH.2.51 | 537 | B | na | na | na | Tick F | Drag | MR | 44.37 | -64.27 | 2006 |
| BH.2.50 | 16 | I | 7A | 10 | 3 | Tick F | Drag | MR | 44.37 | -64.27 | 2006 |
| BH.2.35 | 3 | K | 2A | 3 | 2 | Tick M | Drag | MR | 44.37 | -64.27 | 2006 |
| BH.2.58 | 59 | B | na | na | na | Tick M | Drag | MR | 44.37 | -64.27 | 2006 |
| BH.2.29 | 12 | I | 6A | 9 | 3 | Tick M | Drag | MR | 44.37 | -64.27 | 2006 |
| SR.1.2 | 12 | M | na | na | na | Tick M | Drag | MR | 44.55 | -64.58 | 2006 |
| L.38 | 538 | K | 2A | 3 | 2 | Tick L | WFM | MR | 44.53 | -64.50 | 2006 |
| ER.13 | 12 | M | 6A | 9 | 3 | Tick N | Drag | MR | 43.77 | -65.23 | 2006 |
| CEM.3 | 19 | E | 9 | 19 | 3 | Tick N | Drag | MR | 44.38 | -64.31 | 2006 |
| BH.2.27 | 3 | K | 2A | 3 | 2 | Tick M | Drag | MR | 44.37 | -64.27 | 2006 |
| BH.2 | 3 | K | 2A | 3 | 2 | Tick M | Drag | MR | 44.37 | -64.27 | 2006 |
| L.41 | 4 | H | 2D | 4 | 2 | Tick L | WFM | MR | 44.36 | -64.26 | 2006 |
| L.30 | 1 | A | 1 | 1 | 1 | Tick L | WFM | MR | 44.37 | -64.28 | 2006 |
| L.12 | 59 | B | 3 | 7 | 1 | Tick L | WFM | MR | 44.37 | -64.27 | 2006 |
| CEM.1 | 3 | K | 2A | 3 | 2 | Tick M | Drag | MR | 44.38 | -64.31 | 2006 |
| GM11-004 | 9 | na | 4A | 20 | 2 | Tick N | WFM | MR | 44.70 | -66.82 | 2011 |
| GM11-013 | 36 | na | 4A | 20 | 2 | Tick N | WFM | MR | 44.70 | -66.82 | 2011 |
| GM11-021 | 1 | A | 1 | 1 | 1 | Tick N | WFM | MR | 44.70 | -66.82 | 2011 |
| GM11-030 | 3 | na | 2A | 3 | 2 | Tick L | WFM | MR | 44.70 | -66.82 | 2011 |
| GM11-039 | 1 | na | 1 | 1 | 1 | Tick N | RBV | MR | 44.70 | -66.82 | 2011 |
| Y11-053 | 3 | K | 2A | 3 | 2 | Tick L | ECH | MR | 43.83 | -66.12 | 2011 |
| Y11-057 | 3 | K | 2A | 3 | 2 | Tick L | WFM | MR | 43.83 | -66.12 | 2011 |
| Y11-058 | 3 | K | 2A | 3 | 2 | Tick L | WFM | MR | 43.83 | -66.12 | 2011 |
| GC12-C31 | 19 | E1 | 9 | 19 | 3 | Tick L | ECH | MR | 43.68 | -65.33 | 2012 |
| GC12-C32 | 14 | E | 6B | 18 | 3 | Tick L | ECH | MR | 43.68 | -65.33 | 2012 |
| GC12-DM76 | 1 | A | 1 | 1 | 1 | Tick L | WFM | MR | 43.68 | -65.33 | 2012 |
| GC12-DM118 | 1 | A | 1 | 1 | 1 | Tick L | WFM | MR | 43.68 | -65.33 | 2012 |
| AC12-DM193 | 1 | K | 1 | 1 | 1 | Tick L | WFM | MR | 44.73 | -63.67 | 2012 |
| AC12-DM205 | 1 | A | 1 | 1 | 1 | Tick L | WFM | MR | 44.73 | -63.67 | 2012 |

Table C. A contingency table of the sample sources (host and questing ticks) and the clonal complexes (CC) inferred with single locus variant criterion (SLV).

|  | | | | | | | |
| --- | --- | --- | --- | --- | --- | --- | --- |
| CC at SLV | Tick sources | | | | | | |
|  | DM | ECH | RBV | RSQ | WFM | Ticks | Total |
| CC3 | 0 | 1 | 0 | 1 | 4 | 14 | 20 |
| CC4 | 1 | 0 | 0 | 0 | 1 | 1 | 3 |
| CC7 | 0 | 1 | 0 | 0 | 1 | 2 | 4 |
| CC8 | 0 | 1 | 0 | 0 | 0 | 1 | 2 |
| CC12 | 0 | 1 | 0 | 0 | 0 | 8 | 9 |
| CC16 | 0 | 0 | 0 | 0 | 0 | 4 | 4 |
| CC19 | 0 | 1 | 0 | 0 | 0 | 2 | 3 |
| CC29 | 0 | 1 | 0 | 0 | 0 | 0 | 1 |
| CC34 | 0 | 4 | 0 | 0 | 0 | 1 | 5 |
| CC36 | 0 | 0 | 0 | 0 | 4 | 5 | 9 |
| CC37 | 0 | 0 | 0 | 0 | 0 | 1 | 1 |
| CC48 | 0 | 1 | 0 | 0 | 0 | 0 | 1 |
| CC222 | 1 | 2 | 0 | 0 | 0 | 0 | 3 |
| ST225 | 0 | 0 | 0 | 1 | 0 | 0 | 1 |
| CC228 | 0 | 1 | 0 | 0 | 0 | 0 | 1 |
| ST234 | 0 | 1 | 0 | 0 | 0 | 0 | 1 |
| CC403 | 0 | 0 | 1 | 0 | 7 | 9 | 17 |
| ST519 | 0 | 0 | 0 | 0 | 0 | 1 | 1 |
| ST535 | 0 | 0 | 0 | 0 | 1 | 0 | 1 |
| ST536 | 0 | 0 | 0 | 0 | 1 | 0 | 1 |
| ST538 | 0 | 0 | 0 | 0 | 1 | 0 | 1 |
| ST641 | 0 | 1 | 0 | 0 | 0 | 0 | 1 |
| ST644 | 0 | 0 | 0 | 0 | 0 | 1 | 1 |
| Total | 2 | 16 | 1 | 2 | 20 | 50 | 91 |

Table D. The contingency table of the sample sources (host and questing ticks) and the CCs inferred at double locus variant (DLV).

|  | | | | | | | |
| --- | --- | --- | --- | --- | --- | --- | --- |
| CC at DLV | Tick sources | | | | | | |
|  | DM | ECH | RBV | RSQ | WFM | Ticks | Total |
| CC3 | 1 | 2 | 1 | 1 | 13 | 24 | 42 |
| CC7 | 0 | 1 | 0 | 0 | 1 | 2 | 4 |
| CC12 | 0 | 1 | 0 | 0 | 0 | 8 | 9 |
| CC16 | 0 | 0 | 0 | 0 | 0 | 4 | 4 |
| CC19 | 0 | 1 | 0 | 0 | 0 | 2 | 3 |
| CC29 | 0 | 1 | 0 | 0 | 0 | 0 | 1 |
| CC34 | 0 | 5 | 0 | 0 | 0 | 1 | 6 |
| CC36 | 1 | 2 | 0 | 0 | 4 | 7 | 14 |
| CC37 | 0 | 0 | 0 | 0 | 0 | 1 | 1 |
| ST225 | 0 | 0 | 0 | 0 | 0 | 1 | 1 |
| CC228 | 0 | 0 | 0 | 1 | 0 | 0 | 1 |
| ST234 | 0 | 1 | 0 | 0 | 0 | 0 | 1 |
| ST535 | 0 | 1 | 0 | 0 | 0 | 0 | 1 |
| ST536 | 0 | 0 | 0 | 0 | 1 | 0 | 1 |
| CC641 | 0 | 1 | 0 | 0 | 0 | 0 | 1 |
| ST644 | 0 | 0 | 0 | 0 | 0 | 1 | 1 |
| Total | 2 | 16 | 1 | 2 | 20 | 50 | 91 |

Table E. Summary of the Chi-Square Goodness of Fit Test of the correspondence analysis of the CC at SLV

| Dimension |  | | | | Proportion of Inertia | | Confidence Singular Value | |
| --- | --- | --- | --- | --- | --- | --- | --- | --- |
|  |  |  |  |  |  | |  |  |
|  | Singular Value | Inertia | Chi Square | Sig. | Accounted for | Cumulative | Standard Deviation | Correlation |
| 1 | 0.805 | 0.648 |  |  | 0.373 | 0.373 | 0.058 | 0.065 |
| 2 | 0.715 | 0.511 |  |  | 0.294 | 0.667 | 0.223 |  |
| 3 | 0.564 | 0.318 |  |  | 0.183 | 0.849 |  |  |
| 4 | 0.474 | 0.224 |  |  | 0.129 | 0.978 |  |  |
| 5 | 0.194 | 0.038 |  |  | 0.022 | 1.000 |  |  |
| Total |  | 1.739 | 158.281 | 0.002 | 1.000 | 1.000 |  |  |

Table F. Overview of scores of the different sample sources (host species and questing ticks) in the correspondence analysis of the CC at SLV

| host |  | Score in Dimension | |  | Contribution | | | | |
| --- | --- | --- | --- | --- | --- | --- | --- | --- | --- |
|  |  |  | |  | Of Point to Inertia of Dimension | | Of Dimension to Inertia of Point | | |
|  | Mass | 1 | 2 | Inertia | 1 | 2 | 1 | 2 | Total |
| DM | 0.022 | 1.424 | -0.077 | 0.311 | 0.055 | 0.000 | 0.115 | 0.000 | 0.116 |
| ECH | 0.176 | 1.822 | 0.190 | 0.498 | 0.725 | 0.009 | 0.944 | 0.009 | 0.953 |
| RBV | 0.011 | -0.732 | -0.457 | 0.048 | 0.007 | 0.003 | 0.099 | 0.034 | 0.134 |
| RS | 0.022 | -1.083 | 5.532 | 0.503 | 0.032 | 0.941 | 0.041 | 0.956 | 0.997 |
| WFM | 0.220 | -0.618 | -0.320 | 0.232 | 0.104 | 0.032 | 0.291 | 0.069 | 0.360 |
| Ticks | 0.549 | -0.335 | -0.142 | 0.147 | 0.076 | 0.015 | 0.337 | 0.054 | 0.391 |
| Total | 1.000 |  |  | 1.739 | 1.000 | 1.000 |  |  |  |

Table G. Overview of scores of the different CCs inferred at SLV (at SLV) and singleton STs

| CC at SLV |  | Score in Dimension | |  | Contribution | | | | |
| --- | --- | --- | --- | --- | --- | --- | --- | --- | --- |
|  |  |  | |  | Of Point to Inertia of Dimension | | Of Dimension to Inertia of Point | | |
|  | Mass | 1 | 2 | Inertia | 1 | 2 | 1 | 2 | Total |
| CC3 | 0.22 | -0.399 | 0.172 | 0.044 | 0.043 | 0.009 | 0.634 | 0.105 | 0.739 |
| CC4 | 0.033 | 0.195 | -0.251 | 0.157 | 0.002 | 0.003 | 0.006 | 0.009 | 0.016 |
| CC7 | 0.044 | 0.166 | -0.145 | 0.004 | 0.002 | 0.001 | 0.234 | 0.158 | 0.391 |
| CC8 | 0.022 | 0.923 | 0.034 | 0.019 | 0.023 | 0 | 0.783 | 0.001 | 0.784 |
| CC12 | 0.099 | -0.118 | -0.147 | 0.05 | 0.002 | 0.003 | 0.022 | 0.03 | 0.052 |
| CC16 | 0.044 | -0.416 | -0.198 | 0.036 | 0.009 | 0.002 | 0.17 | 0.034 | 0.204 |
| CC19 | 0.033 | 0.477 | -0.044 | 0.015 | 0.009 | 0 | 0.416 | 0.003 | 0.419 |
| CC29 | 0.011 | 2.262 | 0.266 | 0.052 | 0.07 | 0.001 | 0.879 | 0.011 | 0.89 |
| CC34 | 0.055 | 1.727 | 0.173 | 0.149 | 0.204 | 0.002 | 0.885 | 0.008 | 0.893 |
| CC36 | 0.099 | -0.572 | -0.309 | 0.046 | 0.04 | 0.013 | 0.572 | 0.148 | 0.721 |
| CC37 | 0.011 | -0.416 | -0.198 | 0.009 | 0.002 | 0.001 | 0.17 | 0.034 | 0.204 |
| CC48 | 0.011 | 2.262 | 0.266 | 0.052 | 0.07 | 0.001 | 0.879 | 0.011 | 0.89 |
| CC222 | 0.033 | 2.098 | 0.141 | 0.217 | 0.18 | 0.001 | 0.538 | 0.002 | 0.54 |
| ST225 | 0.011 | -1.345 | 7.738 | 0.489 | 0.025 | 0.92 | 0.033 | 0.962 | 0.995 |
| CC228 | 0.011 | 2.262 | 0.266 | 0.052 | 0.07 | 0.001 | 0.879 | 0.011 | 0.89 |
| ST234 | 0.011 | 2.262 | 0.266 | 0.052 | 0.07 | 0.001 | 0.879 | 0.011 | 0.89 |
| CC403 | 0.187 | -0.59 | -0.327 | 0.111 | 0.081 | 0.028 | 0.469 | 0.128 | 0.598 |
| ST519 | 0.011 | -0.416 | -0.198 | 0.009 | 0.002 | 0.001 | 0.17 | 0.034 | 0.204 |
| ST535 | 0.011 | -0.768 | -0.448 | 0.039 | 0.008 | 0.003 | 0.134 | 0.04 | 0.174 |
| ST536 | 0.011 | -0.768 | -0.448 | 0.039 | 0.008 | 0.003 | 0.134 | 0.04 | 0.174 |
| ST538 | 0.011 | -0.768 | -0.448 | 0.039 | 0.008 | 0.003 | 0.134 | 0.04 | 0.174 |
| ST641 | 0.011 | 2.262 | 0.266 | 0.052 | 0.07 | 0.001 | 0.879 | 0.011 | 0.89 |
| ST644 | 0.011 | -0.416 | -0.198 | 0.009 | 0.002 | 0.001 | 0.17 | 0.034 | 0.204 |
| Total | 1.00 |  |  | 1.739 | 1.00 | 1.00 |  |  |  |

Table H. Summary of the Chi-Square Goodness of Fit Test of the correspondence analysis of the CC at DLV

| Dimension |  | | | | Proportion of Inertia | | Confidence Singular Value | |
| --- | --- | --- | --- | --- | --- | --- | --- | --- |
|  |  |  |  |  |  | |  |  |
|  | Singular Value | Inertia | Chi Square | Sig. | Accounted for | Cumulative | Standard Deviation | Correlation |
| 1 | .722 | .521 |  |  | .439 | .439 | .142 | .965 |
| 2 | .683 | .467 |  |  | .393 | .832 | .119 |  |
| 3 | .412 | .170 |  |  | .143 | .975 |  |  |
| 4 | .146 | .021 |  |  | .018 | .993 |  |  |
| 5 | .093 | .009 |  |  | .007 | 1.000 |  |  |
| Total |  | 1.188 | 108.136 | .007 | 1.000 | 1.000 |  |  |

Table I. Overview of scores of the different CCs (at DLV) and singleton STs

| CC at DLV |  | Score in Dimension | |  | Contribution | | | | |
| --- | --- | --- | --- | --- | --- | --- | --- | --- | --- |
|  |  |  |  |  | Of Point to Inertia of Dimension | | Of Dimension to Inertia of Point | | |
|  | Mass | 1 | 2 | Inertia | 1 | 2 | 1 | 2 | Total |
| CC3 | 0.462 | -0.267 | 0.325 | 0.068 | 0.046 | 0.071 | 0.352 | 0.493 | 0.845 |
| CC7 | 0.044 | 0.267 | -0.063 | 0.004 | 0.004 | 0.000 | 0.541 | 0.028 | 0.570 |
| CC12 | 0.099 | 0.073 | 0.238 | 0.050 | 0.001 | 0.008 | 0.008 | 0.076 | 0.084 |
| CC16 | 0.044 | -0.127 | 0.513 | 0.036 | 0.001 | 0.017 | 0.014 | 0.219 | 0.234 |
| CC19 | 0.033 | 0.473 | -0.312 | 0.015 | 0.010 | 0.005 | 0.366 | 0.150 | 0.516 |
| CC29 | 0.011 | 1.672 | -1.961 | 0.052 | 0.043 | 0.062 | 0.430 | 0.560 | 0.991 |
| CC34 | 0.066 | 1.372 | -1.548 | 0.198 | 0.172 | 0.231 | 0.453 | 0.546 | 0.999 |
| CC36 | 0.154 | 0.061 | 0.213 | 0.027 | 0.001 | 0.010 | 0.015 | 0.177 | 0.193 |
| CC37 | 0.011 | -0.127 | 0.513 | 0.009 | 0.000 | 0.004 | 0.014 | 0.219 | 0.234 |
| ST225 | 0.011 | -6.233 | -4.900 | 0.489 | 0.591 | 0.386 | 0.630 | 0.369 | 0.999 |
| CC228 | 0.011 | 1.672 | -1.961 | 0.052 | 0.043 | 0.062 | 0.430 | 0.560 | 0.991 |
| ST234 | 0.011 | 1.672 | -1.961 | 0.052 | 0.043 | 0.062 | 0.430 | 0.560 | 0.991 |
| ST535 | 0.011 | -0.351 | 0.683 | 0.039 | 0.002 | 0.008 | 0.025 | 0.090 | 0.115 |
| ST536 | 0.011 | -0.351 | 0.683 | 0.039 | 0.002 | 0.008 | 0.025 | 0.090 | 0.115 |
| CC641 | 0.011 | 1.672 | -1.961 | 0.052 | 0.043 | 0.062 | 0.430 | 0.560 | 0.991 |
| ST644 | 0.011 | -0.127 | 0.513 | 0.009 | 0.000 | 0.004 | 0.014 | 0.219 | 0.234 |
| Total | 1.000 |  |  | 1.188 | 1.000 | 1.000 |  |  |  |

Table J. Overview of scores of the different sample sources (host species and questing ticks) with CCs inferred at DLV.

| Host |  | Score in Dimension | |  | Contribution | | | | |
| --- | --- | --- | --- | --- | --- | --- | --- | --- | --- |
|  |  |  | |  | Of Point to Inertia of Dimension | | Of Dimension to Inertia of Point | | |
|  | Mass | 1 | 2 | Inertia | 1 | 2 | 1 | 2 | Total |
| DM | 0.022 | -0.143 | 0.394 | 0.026 | 0.001 | 0.005 | 0.013 | 0.091 | 0.103 |
| ECH | 0.176 | 1.207 | -1.340 | 0.402 | 0.355 | 0.462 | 0.460 | 0.537 | 0.997 |
| RBV | 0.011 | -0.370 | 0.476 | 0.013 | 0.002 | 0.004 | 0.085 | 0.133 | 0.217 |
| RS | 0.022 | -4.501 | -3.348 | 0.490 | 0.617 | 0.361 | 0.656 | 0.344 | 1.000 |
| WFM | 0.220 | -0.254 | 0.467 | 0.151 | 0.020 | 0.070 | 0.068 | 0.217 | 0.284 |
| Ticks | 0.549 | -0.092 | 0.351 | 0.107 | 0.006 | 0.099 | 0.031 | 0.431 | 0.462 |
| Total | 1.000 |  |  | 1.188 | 1.000 | 1.000 |  |  |  |

Table K. Associations of host species with STs of CC34 at SLV. The models did not include of visits as a random effect because this was not significant, but did include site ID nested by region as a random effect because this was significant (β = 0.03 ± 0.01; Wald-test = 1.8; *P =* 0.0358 corresponding to 49.6 % of the total variation). Reducing the parameters from the full model to the minimal model including questing ticks did not significantly affect model deviance (χ^2^ = 4.18, df = 3, P >0.1).

| **Type III Tests of Fixed Effects** | | | | | | | | | |
| --- | --- | --- | --- | --- | --- | --- | --- | --- | --- |
| Factor | Num DF | | Den DF | | F Value | | | *P* > F | |
| Host | 5 | | 63 | | 4.28 | | | 0.002 | |
| **Full model** | | | | | | | | | |
| Factor | | Estimate | | Standard Error | | DF | t Value | | P > \|t\| |
| Intercept | | 0.01320 | | 0.07624 | | 22 | 0.17 | | 0.864 |
| DM | | 0.07628 | | 0.1655 | | 63 | 0.46 | | 0.646 |
| ECH | | 0.3340 | | 0.09421 | | 63 | 3.55 | | 0.001 |
| RBV | | -0.0237 | | 0.2311 | | 63 | -0.10 | | 0.918 |
| RS | | 0.06330 | | 0.1556 | | 63 | 0.41 | | 0.685 |
| WFM | | -0.0344 | | 0.07780 | | 63 | -0.44 | | 0.659 |
| Questing ticks: reference | |  | |  | |  |  | |  |
| **Minimal model with questing ticks** | | | | | | | | | |
| Factor | | Estimate | | Standard Error | | DF | t Value | | P > \|t\| |
| Intercept | | 0.01298 | | 0.07381 | | 22 | 0.18 | | 0.862 |
| ECH | | 0.3207 | | 0.09097 | | 66 | 3.53 | | <0.001 |
| Other host spp. | | -0.0146 | | 0.07325 | | 66 | -0.20 | | 0.843 |
| Questing ticks: reference | |  | |  | |  |  | |  |
| **Minimal model without questing ticks** | | | | | | | | | |
| Factor | | Estimate | | Standard Error | | DF | t Value | | P > \|t\| |
| Intercept | | 0 | | 0.05547 | | 39 | 0.00 | | 1.0 |
| ECH | | 0.2500 | | 0.089 | | 39 | 2.82 | | 0.008 |
| Other host spp.: reference | |  | |  | |  |  | |  |

Table L. Associations of host species with STs of CC34 at DLV. The models did not include of visits as a random effect because this was not significant, but did include site ID nested by region as a random effect because this was significant (β = 0.04 ± 0.02; Wald-test = 2.07; *P =* 0.0192 corresponding to 55.4 % of the total variation). Reducing the parameters from the full model to the minimal model including questing ticks did not significantly affect model deviance (χ^2^ = 3.06, df = 3, P >0.1).

| **Type III Tests of Fixed Effects** | | | | | | | | | |
| --- | --- | --- | --- | --- | --- | --- | --- | --- | --- |
| Factor | Num DF | | Den DF | | F Value | | | *P* > F | |
| Host | 5 | | 63 | | 7.00 | | | <0.001 | |
| **Full model** | | | | | | | | | |
| Factor | | Estimate | | Standard Error | | DF | t Value | | P > \|t\| |
| Intercept | | 0.01873 | | 0.08054 | | 22 | 0.23 | | 0.818 |
| DM | | -0.1672 | | 0.1689 | | 63 | -0.99 | | 0.326 |
| ECH | | 0.4267 | | 0.09729 | | 63 | 4.39 | | <0.001 |
| RBV | | -0.0247 | | 0.2366 | | 63 | -0.10 | | 0.917 |
| RS | | 0.1187 | | 0.1576 | | 63 | 0.75 | | 0.454 |
| WFM | | -0.0296 | | 0.07999 | | 63 | -0.37 | | 0.713 |
| Questing ticks: reference | |  | |  | |  |  | |  |
| **Minimal model with questing ticks** | | | | | | | | | |
| Factor | | Estimate | | Standard Error | | DF | t Value | | P > \|t\| |
| Intercept | | 0.01951 | | 0.07786 | | 22 | 0.25 | | 0.805 |
| ECH | | 0.4083 | | 0.09428 | | 66 | 4.33 | | <0.001 |
| Other host spp. | | -0.0287 | | 0.07564 | | 66 | -0.38 | | 0.705 |
| Questing ticks: reference | |  | |  | |  |  | |  |
| **Minimal model without questing ticks** | | | | | | | | | |
| Factor | | Estimate | | Standard Error | | DF | t Value | | P > \|t\| |
| Intercept | | 0 | | 0.05938 | | 39 | 0.00 | | 1.0 |
| ECH | | 0.3125 | | 0.09505 | | 39 | 3.29 | | 0.002 |
| Other host spp.: reference | |  | |  | |  |  | |  |

Table M. Associations of host species with STs of CC403 at SLV. The random effects were not significant and not included in the models. Reducing the parameters from the full model to the minimal model including questing ticks did not significantly affect model deviance (χ^2^ = 5.22, df = 3, P >0.1).

| **Type III Tests of Fixed Effects** | | | | | | | | | |
| --- | --- | --- | --- | --- | --- | --- | --- | --- | --- |
| Factor | Num DF | | Den DF | | F Value | | | *P* > F | |
| Host | 5 | | 85 | | 2.70 | | | 0.026 | |
| **Full model** | | | | | | | | | |
| Factor | | Estimate | | Standard Error | | DF | t Value | | P > \|t\| |
| Intercept | | 0.1800 | | 0.05298 | | 85 | 3.40 | | 0.001 |
| DM | | -0.1800 | | 0.2702 | | 85 | -0.67 | | 0.507 |
| ECH | | -0.1800 | | 0.1076 | | 85 | -1.67 | | 0.098 |
| RBV | | 0.8200 | | 0.3784 | | 85 | 2.17 | | 0.033 |
| RS | | -0.1800 | | 0.2702 | | 85 | -0.67 | | 0.507 |
| WFM | | 0.3500 | | 0.08377 | | 85 | 4.18 | | <0.001 |
| Questing ticks: reference | |  | |  | |  |  | |  |
| **Minimal model with questing ticks** | | | | | | | | | |
| Factor | | Estimate | | Standard Error | | DF | t Value | | P > \|t\| |
| Intercept | | 0.1800 | | 0.05411 | | 88 | 3.33 | | 0.001 |
| WFM | | 0.3500 | | 0.08555 | | 88 | 4.09 | | <0.001 |
| Other host spp. | | 0.04762 | | 0.08349 | | 88 | 0.57 | | 0.570 |
| Questing ticks: reference | |  | |  | |  |  | |  |
| **Minimal model without questing ticks** | | | | | | | | | |
| Factor | | Estimate | | Standard Error | | DF | t Value | | P > \|t\| |
| Intercept | | 0.04762 | | 0.08197 | | 39 | 0.58 | | 0.565 |
| WFM | | 0.3024 | | 0.1174 | | 39 | 2.58 | | 0.014 |
| Other host spp.: reference | |  | |  | |  |  | |  |

Table N. Associations of host species with STs of CC4 at SLV. The models did not include visits as a random effect because this was not significant, but did include site ID nested by region as a random effect because this was significant (β = 0.027 ± 0.014; Wald-test = 1.9; *P =* 0.0312 corresponding to 58.2 % of the total variation). Reducing the parameters from the full model to the minimal model including questing ticks did not significantly affect model deviance (χ^2^ = 6.97, df = 3, P >0.05).

| **Type III Tests of Fixed Effects** | | | | | | | | | |
| --- | --- | --- | --- | --- | --- | --- | --- | --- | --- |
| Factor | Num DF | | Den DF | | F Value | | | *P* > F | |
| Host | 5 | | 63 | | 3.21 | | | 0.012 | |
| **Full model** | | | | | | | | | |
| Factor | | Estimate | | Standard Error | | DF | t Value | | P > \|t\| |
| Intercept | | 0.06703 | | 0.06409 | | 22 | 1.05 | | 0.307 |
| DM | | 0.4501 | | 0.1321 | | 63 | 3.41 | | 0.001 |
| ECH | | -0.0751 | | 0.07650 | | 63 | -0.98 | | 0.329 |
| RBV | | -0.0323 | | 0.1854 | | 63 | -0.17 | | 0.862 |
| RS | | -0.0557 | | 0.1228 | | 63 | -0.45 | | 0.652 |
| WFM | | -0.0074 | | 0.06277 | | 63 | -0.12 | | 0.907 |
| Questing ticks: reference | |  | |  | |  |  | |  |
| **Minimal model with questing ticks** | | | | | | | | | |
| Factor | | Estimate | | Standard Error | | DF | t Value | | P > \|t\| |
| Intercept | | 0.06157 | | 0.06311 | | 22 | 0.98 | | 0.340 |
| DM | | 0.4662 | | 0.1296 | | 66 | 3.60 | | <0.001 |
| Other host spp. | | -0.0279 | | 0.05841 | | 66 | -0.48 | | 0.633 |
| Questing ticks: reference | |  | |  | |  |  | |  |
| **Minimal model without questing ticks** | | | | | | | | | |
| Factor | | Estimate | | Standard Error | | DF | t Value | | P > \|t\| |
| Intercept | | 0.02564 | | 0.03113 | | 39 | 0.82 | | 0.564 |
| DM | | 0.4744 | | 0.1410 | | 39 | 3.37 | | 0.014 |
| Other host spp.: reference | |  | |  | |  |  | |  |

Table O. Associations of host species with RST1 type IGS sequences. The models did not include of visits as a random effect because this was not significant, but did include site ID nested by region as a random effect because this was significant (β = 0.026 ± 0.02; Wald-test = 1.07; *P =* 0.1426). Reducing the parameters from the full model to the minimal model including questing ticks did not significantly affect model deviance (χ^2^ = 5.01, df = 3, P >0.1).

| **Type III Tests of Fixed Effects** | | | | | | | | | |
| --- | --- | --- | --- | --- | --- | --- | --- | --- | --- |
| Factor | Num DF | | Den DF | | F Value | | | *P* > F | |
| Host | 5 | | 74 | | 2.38 | | | 0.046 | |
| **Full model** | | | | | | | | | |
| Factor | | Estimate | | Standard Error | | DF | t Value | | P > \|t\| |
| Intercept | | 0.1905 | | 0.05956 | | 74 | 3.20 | | 0.002 |
| DM | | -0.1905 | | 0.2794 | | 74 | -0.68 | | 0.497 |
| ECH | | -0.1905 | | 0.1225 | | 74 | -1.55 | | 0.124 |
| RBV | | 0.8095 | | 0.3906 | | 74 | 2.07 | | 0.042 |
| RS | | -0.1905 | | 0.2794 | | 74 | -0.68 | | 0.497 |
| WFM | | 0.3500 | | 0.08631 | | 74 | 4.05 | | <0.001 |
| Questing ticks: reference | |  | |  | |  |  | |  |
| **Minimal model with questing ticks** | | | | | | | | | |
| Factor | | Estimate | | Standard Error | | DF | t Value | | P > \|t\| |
| Intercept | | 0.1905 | | 0.06084 | | 77 | 3.13 | | 0.002 |
| WFM | | 0.3500 | | 0.08817 | | 77 | 3.97 | | <0.001 |
| Other host spp. | | 0.05556 | | 0.09293 | | 77 | 0.60 | | 0.552 |
| Questing ticks: reference | |  | |  | |  |  | |  |
| **Minimal model without questing ticks** | | | | | | | | | |
| Factor | | Estimate | | Standard Error | | DF | t Value | | P > \|t\| |
| Intercept | | 0.05556 | | 0.09208 | | 36 | 0.60 | | 0.550 |
| WFM | | 0.2944 | | 0.1269 | | 36 | 2.32 | | 0.026 |
| Other host spp.: reference | |  | |  | |  |  | |  |

Table P. Associations of host species with RST2 type IGS sequences (IGS4). The random effects were not significant and not included in the model. Reducing the parameters from the full model to the minimal model including questing ticks did not significantly affect model deviance (χ^2^ = 3.64, df = 3, P >0.1).

| **Type III Tests of Fixed Effects** | | | | | | | | | |
| --- | --- | --- | --- | --- | --- | --- | --- | --- | --- |
| Factor | Num DF | | Den DF | | F Value | | | *P* > F | |
| Host | 5 | | 74 | | 4.55 | | | 0.001 | |
| **Full model** | | | | | | | | | |
| Factor | | Estimate | | Standard Error | | DF | t Value | | P > \|t\| |
| Intercept | | 0.02381 | | 0.03696 | | 74 | 0.64 | | 0.521 |
| DM | | 0.4762 | | 0.1734 | | 74 | 2.75 | | 0.007 |
| ECH | | 0.2839 | | 0.07602 | | 74 | 3.73 | | <0.001 |
| RBV | | -0.0238 | | 0.2424 | | 74 | -0.10 | | 0.922 |
| RS | | -0.0238 | | 0.1734 | | 74 | -0.14 | | 0.891 |
| WFM | | -0.0238 | | 0.06507 | | 74 | -0.37 | | 0.715 |
| Questing ticks: reference | |  | |  | |  |  | |  |
| **Minimal model with questing ticks** | | | | | | | | | |
| Factor | | Estimate | | Standard Error | | DF | t Value | | P > \|t\| |
| Intercept | | 0.02381 | | 0.03814 | | 77 | 0.62 | | 0.534 |
| ECH | | 0.2839 | | 0.07846 | | 77 | 3.62 | | <0.001 |
| Other host spp. | | 0.01619 | | 0.06244 | | 77 | 0.26 | | 0.796 |
| Questing ticks: reference | |  | |  | |  |  | |  |
| **Minimal model without questing ticks** | | | | | | | | | |
| Factor | | Estimate | | Standard Error | | DF | t Value | | P > \|t\| |
| Intercept | | 0.04000 | | 0.06437 | | 36 | 0.62 | | 0.538 |
| ECH | | 0.2677 | | 0.1101 | | 36 | 2.43 | | 0.020 |
| Other host spp.: reference | |  | |  | |  |  | |  |

Table Q. Associations of host species with *ospC* G. The random effect of number of visits was significant and included in the model (β = 0.025 ± 0.009; Wald-test = 2.76; *P =* 0.0029 corresponding to 74.6 % of the total variation), but the random effect of site ID nested by region was not significant and not included in the model. Reducing the parameters from the full model to the minimal model including questing ticks did not significantly affect model deviance (χ^2^ = 2.88, df = 2, P >0.1).

| **Type III Tests of Fixed Effects** | | | | | | | | | |
| --- | --- | --- | --- | --- | --- | --- | --- | --- | --- |
| Factor | Num DF | | Den DF | | F Value | | | *P* > F | |
| Host | 4 | | 33 | | 5.67 | | | 0.001 | |
| **Full model** | | | | | | | | | |
| Factor | | Estimate | | Standard Error | | DF | t Value | | P > \|t\| |
| Intercept | | -0.00902 | | 0.05113 | | 31 | -0.18 | | 0.861 |
| DM | | 0.009021 | | 0.1449 | | 33 | 0.06 | | 0.951 |
| ECH | | 0.2716 | | 0.07427 | | 33 | 3.66 | | <0.001 |
| RS | | 0.1447 | | 0.1207 | | 33 | 1.20 | | 0.239 |
| WFM | | -0.02032 | | 0.05768 | | 33 | -0.35 | | 0.727 |
| Questing ticks: reference | |  | |  | |  |  | |  |
| **Minimal model with questing ticks** | | | | | | | | | |
| Factor | | Estimate | | Standard Error | | DF | t Value | | P > \|t\| |
| Intercept | | -0.00216 | | 0.04874 | | 32 | -0.04 | | 0.965 |
| ECH | | 0.2471 | | 0.07165 | | 34 | 3.45 | | 0.002 |
| Other host spp. | | -0.00637 | | 0.05550 | | 34 | -0.11 | | 0.909 |
| Questing ticks: reference | |  | |  | |  |  | |  |
| **Minimal model without questing ticks** | | | | | | | | | |
| Factor | | Estimate | | Standard Error | | DF | t Value | | P > \|t\| |
| Intercept | | -0.00161 | | 0.05148 | | 5 | -0.03 | | 0.976 |
| ECH | | 0.2113 | | 0.07590 | | 5 | 2.78 | | 0.039 |
| Other host spp.: reference | |  | |  | |  |  | |  |

Table R. Associations of host species with *ospC* A. The random effects were not significant and not included in the model. Reducing the parameters from the full model to the minimal model including questing ticks did not significantly affect model deviance (χ^2^ = 0.88, df = 2, P >0.1).

| **Type III Tests of Fixed Effects** | | | | | | | | | |
| --- | --- | --- | --- | --- | --- | --- | --- | --- | --- |
| Factor | Num DF | | Den DF | | F Value | | | *P* > F | |
| Host | 4 | | 64 | | 4.73 | | | 0.002 | |
| **Full model** | | | | | | | | | |
| Factor | | Estimate | | Standard Error | | DF | t Value | | P > \|t\| |
| Intercept | | 0.2632 | | 0.06802 | | 64 | 3.87 | | 0.003 |
| DM | | 0.01503 | | 0.2942 | | 64 | 0.05 | | 0.960 |
| ECH | | -0.0189 | | 0.1345 | | 64 | -0.14 | | 0.889 |
| RS | | 0.02967 | | 0.2910 | | 64 | 0.10 | | 0.919 |
| WFM | | 0.3525 | | 0.1040 | | 64 | 3.39 | | 0.002 |
| Questing ticks: reference | |  | |  | |  |  | |  |
| **Minimal model with questing ticks** | | | | | | | | | |
| Factor | | Estimate | | Standard Error | | DF | t Value | | P > \|t\| |
| Intercept | | 0.2632 | | 0.06698 | | 66 | 3.93 | | 0.002 |
| WFM | | 0.3529 | | 0.1001 | | 66 | 3.52 | | 0.008 |
| Other host spp. | | -0.2632 | | 0.1291 | | 66 | -2.04 | | 0.050 |
| Questing ticks: reference | |  | |  | |  |  | |  |
| **Minimal model without questing ticks** | | | | | | | | | |
| Factor | | Estimate | | Standard Error | | DF | t Value | | P > \|t\| |
| Intercept | | 0.00002 | | 0.09779 | | 29 | 0.00 | | 1.0 |
| WFM | | 0.3529 | | 0.1321 | | 29 | 2.67 | | 0.012 |
| Other host spp.: reference | |  | |  | |  |  | |  |

Table S. Associations of host species with *ospC* H. The random effects were not significant and not included in the model. Reducing the parameters from the full model to the minimal model including questing ticks did not significantly affect model deviance (χ^2^ = 1.19, df = 2, P >0.1).

| **Type III Tests of Fixed Effects** | | | | | | | | | |
| --- | --- | --- | --- | --- | --- | --- | --- | --- | --- |
| Factor | Num DF | | Den DF | | F Value | | | *P* > F | |
| Host | 4 | | 64 | | 2.62 | | | 0.043 | |
| **Full model** | | | | | | | | | |
| Factor | | Estimate | | Standard Error | | DF | t Value | | P > \|t\| |
| Intercept | | 0.02632 | | 0.03649 | | 64 | 0.72 | | 0.473 |
| DM | | 0.4737 | | 0.1632 | | 64 | 2.90 | | 0.005 |
| ECH | | -0.0263 | | 0.07995 | | 64 | -0.33 | | 0.743 |
| RS | | -0.0263 | | 0.1632 | | 64 | -0.16 | | 0.872 |
| WFM | | 0.09133 | | 0.06564 | | 64 | 1.39 | | 0.167 |
| Questing ticks: reference | |  | |  | |  |  | |  |
| **Minimal model with questing ticks** | | | | | | | | | |
| Factor | | Estimate | | Standard Error | | DF | t Value | | P > \|t\| |
| Intercept | | 0.02632 | | 0.03647 | | 66 | 0.72 | | 0.473 |
| DM | | 0.4737 | | 0.1631 | | 66 | 2.90 | | 0.005 |
| Other host spp. | | 0.04265 | | 0.05543 | | 66 | 0.77 | | 0.444 |
| Questing ticks: reference | |  | |  | |  |  | |  |
| **Minimal model without questing ticks** | | | | | | | | | |
| Factor | | Estimate | | Standard Error | | DF | t Value | | P > \|t\| |
| Intercept | | 0.06897 | | 0.05300 | | 29 | 1.30 | | 0.203 |
| DM | | 0.4310 | | 0.2086 | | 29 | 2.07 | | 0.048 |
| Other host spp.: reference | |  | |  | |  |  | |  |

Table T. Comparisons of the minimal models (with questing ticks) presented in Tables S4a to S4i and the respective intercept-only models.

| Outcome | Model | | -2 Log Likelihood | DF | chisq | *P* > \|chisq\| |
| --- | --- | --- | --- | --- | --- | --- |
| CC34S | Minimal Model |  | 8.67 | 1 | 12.58 | <0.001 |
|  | Intercept Model |  | 21.25 |  |  |  |
| CC34D | Minimal Model |  | 10.06 | 1 | 6.42 | 0.011 |
|  | Intercept Model |  | 3.64 |  |  |  |
| CC403S | Minimal Model |  | 86.76 | 2 | 6.42 | 0.040 |
|  | Intercept Model |  | 80.34 |  |  |  |
| CC4S | Minimal Model |  | 49.21 | 1 | 12.44 | <0.001 |
|  | Intercept Model |  | 61.65 |  |  |  |
| RST1 | Minimal Model |  | 94.58 | 2 | 9.02 | 0.011 |
|  | Intercept Model |  | 85.56 |  |  |  |
| RST2 (IGS4) | Minimal Model |  | 19.78 | 2 | 6.96 | 0.031 |
|  | Intercept Model |  | 12.82 |  |  |  |
| *ospC* G | Minimal Model |  | 56.85 | 1 | 17.44 | <0.001 |
|  | Intercept Model |  | 74.29 |  |  |  |
| *ospC* A | Minimal Model |  | 76.77 | 2 | 6.1 | 0.047 |
|  | Intercept Model |  | 70.67 |  |  |  |
| *ospC* H | Minimal Model |  | 0.49 | 1 | 3.94 | 0.047 |
|  | Intercept Model |  | 4.43 |  |  |  |

Table U. Statistics of the minimal models with questing tick data in Tables S4a-i when using GLMs in R software.

| **CC34S with region/SitesID random effect** | | | | |
| --- | --- | --- | --- | --- |
| Factor | Estimate | Standard Error | Z Value | *P* > \|z\| |
| Intercept | -4.053 | 1.133 | -3.578 | <0.001 |
| ECH | 2.601 | 1.282 | 2.028 | 0.042 |
| Other host spp. | -0.2677 | < 0.0001 | 0.000 | 0.999 |
| Questing ticks: reference |  |  |  |  |
| **CC34D with region/SitesID random effect** | | | | |
| Factor | Estimate | Standard Error | Z Value | *P* > \|z\| |
| Intercept | -4.039 | 1.123 | -3.595 | <0.001 |
| ECH | 2.895 | 1.252 | 2.312 | 0.021 |
| Other host spp. | -0.3084 | 0.0000008 | 0.000 | 0.999 |
| Questing ticks: reference |  |  |  |  |
| **CC403S** | | | | |
| Factor | Estimate | Standard Error | t Value | *P* > \|z\| |
| Intercept | 0.18000 | 0.05411 | 3.327 | 0.001 |
| WFM | 0.17000 | 0.09949 | 1.679 | 0.097 |
| Other host spp. | -0.13238 | 0.10123 | -1.331 | 0.187 |
| Questing ticks: reference |  |  |  |  |
| **CC4S with region/SitesID random effect** | | | | |
| Factor | Estimate | Standard Error | Z Value | *P* > \|z\| |
| Intercept | -3.8918 | 1.0102 | -3.853 | <0.001 |
| DM | 3.8918 | 1.7379 | 2.239 | 0.025 |
| Other host spp. | 0.2542 | 1.4306 | 0.178 | 0.859 |
| Questing ticks: reference |  |  |  |  |
| **RST 1** | | | | |
| Factor | Estimate | Standard Error | t Value | *P* > \|z\| |
| Intercept | 0.21429 | 0.06378 | 3.360 | 0.001 |
| WFM | 0.21429 | 0.11047 | 1.940 | 0.056 |
| Other host spp. | -0.15546 | 0.11882 | -1.308 | 0.194 |
| Questing ticks: reference |  |  |  |  |
| **RST2 (IGS4)** | | | | |
| Factor | Estimate | Standard Error | t Value | *P* > \|z\| |
| Intercept | 0.02381 | 0.03814 | 0.624 | 0.534 |
| ECH | 0.28388 | 0.07846 | 3.61 | <0.001 |
| Other host spp. | 0.01619 | 0.06244 | 0.259 | 0.796 |
| Questing ticks: reference |  |  |  |  |

Table U continued.

| ***ospC* G with number of visits random effect** | | | | |
| --- | --- | --- | --- | --- |
| Factor | Estimate | Standard Error | z Value | *P* > \|z\| |
| Intercept | 0.00002673 | 0.02526 | 0.000 | 1.0 |
| ECH | 0.2 | 0.05534 | 3.614 | <0.001 |
| Other host spp. | -0.0000302 | 0.04234 | 0.000 | 1.0 |
| Questing ticks: reference |  |  |  |  |
| ***ospC* A** | | | | |
| Factor | Estimate | Standard Error | t Value | *P* > \|z\| |
| Intercept | 0.2632 | 0.1291 | 2.039 | 0.045 |
| WFM | 0.3529 | 0.149 | 2.369 | 0.021 |
| Other host spp. | 0.00000052 | 0.01103 | 0.000 | 1.0 |
| Questing ticks: reference |  |  |  |  |
| ***ospC* H** | | | | |
| Factor | Estimate | Standard Error | t Value | *P* > \|z\| |
| Intercept | 0.02632 | 0.03647 | 0.722 | 0.473 |
| DM | 0.47368 | 0.16310 | 2.904 | 0.005 |
| Other host spp. | 0.04265 | 0.05543 | 0.769 | 0.444 |
| Questing ticks: reference |  |  |  |  |

Table V. Statistics of the minimal models without questing tick data in Tables S4a-i when using GLMs in R software.

| **CC34S with region/SitesID random effect** | | | | |
| --- | --- | --- | --- | --- |
| Factor | Estimate | Standard Error | Z Value | *P* > \|z\| |
| Intercept | -0.00001 | 0.005547 | 0.000 | 1.0 |
| ECH | 0.2500 | 0.008880 | 2.815 | 0.008 |
| Other host spp. reference |  |  |  |  |
| **CC34D with region/SitesID random effect** | | | | |
| Factor | Estimate | Standard Error | Z Value | *P* > \|z\| |
| Intercept | -0.00001 | 0.005938 | 0.000 | 1.0 |
| ECH | 0.03125 | 0.009505 | 3.288 | 0.002 |
| Other host spp. reference |  |  |  |  |
| **CC403S** | | | | |
| Factor | Estimate | Standard Error | t Value | *P* > \|z\| |
| Intercept | 0.04762 | 0.08197 | 0.581 | 0.565 |
| WFM | 0.30238 | 0.11736 | 2.577 | 0.014 |
| Other host spp. reference |  |  |  |  |
| **CC4S with region/SitesID random effect** | | | | |
| Factor | Estimate | Standard Error | Z Value | *P* > \|z\| |
| Intercept | 0.02564 | 0.03113 | 0.824 | 0.415 |
| DM | 0.47436 | 0.14097 | 3.365 | 0.002 |
| Other host spp. reference |  |  |  |  |
| **RST 1** | | | | |
| Factor | Estimate | Standard Error | t Value | *P* > \|z\| |
| Intercept | 0.1111 | 0.1008 | 1.103 | 0.277 |
| WFM | 0.2889 | 0.1389 | 2.080 | 0.045 |
| Other host spp. reference |  |  |  |  |
| **RST2 (IGS4)** | | | | |
| Factor | Estimate | Standard Error | t Value | *P* > \|z\| |
| Intercept | 0.04000 | 0.06437 | 0.621 | 0.538 |
| ECH | 0.26769 | 0.11005 | 2.432 | 0.020 |
| Other host spp. reference |  |  |  |  |

Table V continued.

| ***ospC* G with number of visits random effect** | | | | |
| --- | --- | --- | --- | --- |
| Factor | Estimate | Standard Error | z Value | *P* > \|z\| |
| Intercept | 0.00000003 | 0.005126 | 0.000 | 1.0 |
| ECH | 0.02 | 0.009025 | 2.216 | 0.035 |
| Other host spp. reference |  |  |  |  |
| ***ospC* A** | | | | |
| Factor | Estimate | Standard Error | t Value | *P* > \|z\| |
| Intercept | 0.00008 | 0.009779 | 0.000 | 1.0 |
| WFM | 0.03529 | 0.0 1321 | 2.673 | 0.012 |
| Other host spp. reference |  |  |  |  |
| ***ospC* H** | | | | |
| Factor | Estimate | Standard Error | t Value | *P* > \|z\| |
| Intercept | 0.06897 | 0.05300 | 1.301 | 0.203 |
| DM | 0.43103 | 0.20865 | 2.066 | 0.045 |
| Other host spp. reference |  |  |  |  |
