# Supplementary material for: Evidence for Host-Genotype Associations of Borrelia burgdorferi Sensu Stricto
Source: PLoS One. 2016 Feb 22;11(2):e0149345. doi: 10.1371/journal.pone.0149345 (PMC4763156; doi:10.1371/journal.pone.0149345)
Supplement: S2 File — These comprise an unrooted Bayesian phylogenetic tree and a minimum spanning tree of the MLST STs. (DOCX) [file pone.0149345.s002.docx]

Fig. A

Unrooted Bayesian phylogenetic tree based on STs constructed without outgroup and the corresponding clonal complexes (CCs) at single locus variant (SLV) and double locus variant (DLV) performed using goeburst. The posterior probabilities produced by the tree are used to support the confidence level of each CC


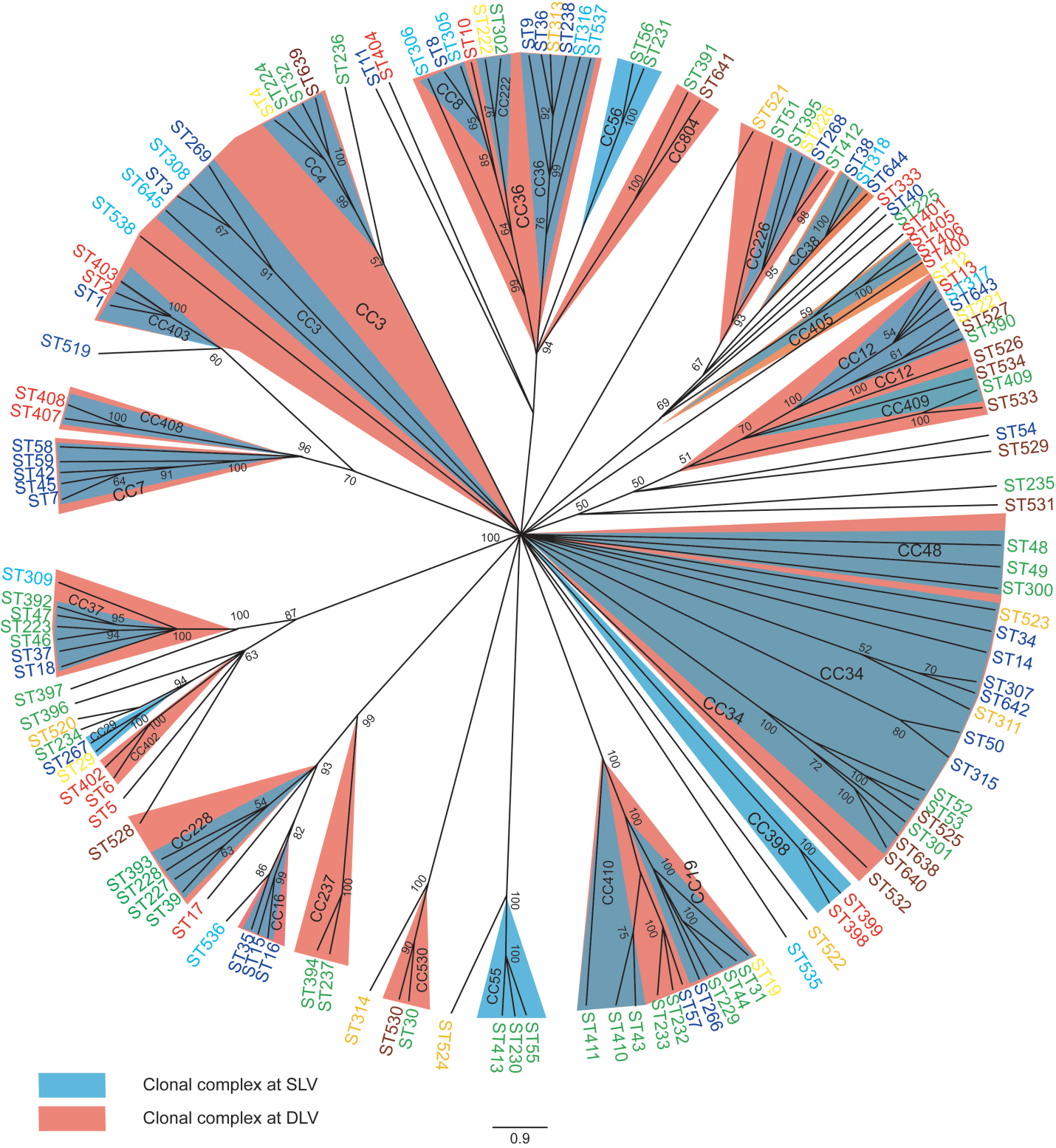


Fig. B. A minimum spanning tree of all the STs in the MLSTNet database created in GoeBURST. The STs are colour coded according to their geographic region of origin: blue, STs found in the ‘northeast’ (i.e. STs found in Northeastern US and also in Quebec, eastern Ontario and the Maritimes); green, STs found in the ‘midwest’ (i.e. STs found in Midwestern US and also in Manitoba); yellow, STs occurring in both the ‘northeast’ and the ‘midwest’; red, STs found in California; cyan STs found only in the Maritimes; orange, STs found only at Long Point Ontario; brown, STs found only in Manitoba. Lines connecting the STs indicate the numbers of different loci between the connected STs: black lines = SLV, gray lines = DLV, dashed lines = TLV and greater. For differences > TLV the optimal phylogenetic edge produced by GoeBURST was the potential ancestry link. Statistics presented for each link are number of locus differences and the spanning edge betweenness statistic. High values at or near 100% indicate that the predicted link is unique while low values indicate that while the predicted link is the optimal one, there are many other pathways from ancestor to descendant, which may be associated with horizontal gene transfer [1]


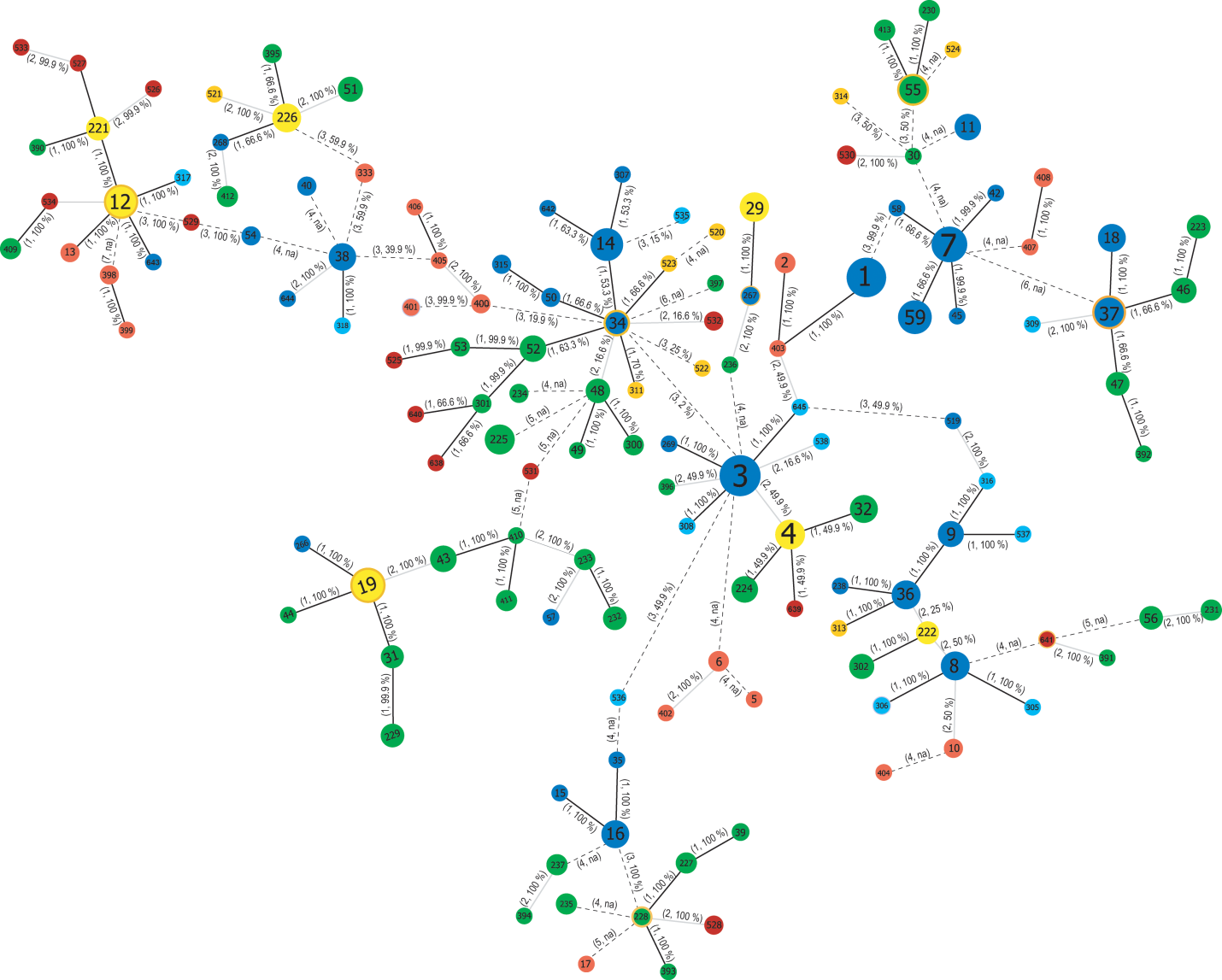


**References**

1. Teixeira AS, Monteiro PT, Carriço JA, Ramirez M, Francisco AP. Not seeing the forest for the trees: size of the minimum spanning trees (MSTs) forest and branch significance in MST-based phylogenetic analysis. PLoS One. 2015;10(3): e0119315.
